# Pectic homogalacturonan sensed by *Bacillus* acts as host associated cue to promote establishment and persistence in the rhizosphere

**DOI:** 10.1016/j.isci.2023.107925

**Published:** 2023-09-15

**Authors:** Farah Boubsi, Grégory Hoff, Anthony Arguelles Arias, Sébastien Steels, Sofija Andrić, Adrien Anckaert, Romain Roulard, Augustin Rigolet, Olivier van Wuytswinkel, Marc Ongena

**Affiliations:** 1 Microbial Processes and Interactions, TERRA Teaching and Research Center, University of Liège - Gembloux Agro-Bio Tech, 5030 Gembloux, Belgium; 2 UMRT INRAe 1158 Plant Biology and Innovation, University of Picardie Jules Verne, UFR des Sciences, 80039 Amiens, France

**Keywords:** Plant biology, Process in plant, Plant development

## Abstract

*Bacillus velezensis* isolates are among the most promising plant-associated beneficial bacteria used as biocontrol agents. However, various aspects of the chemical communication between the plant and these beneficials, determining root colonization ability, remain poorly described. Here we investigated the molecular basis of such interkingdom interaction occurring upon contact between *Bacillus velezensis* and its host via the sensing of pectin backbone homogalacturonan (HG). We showed that *B*. *velezensis* stimulates key developmental traits via a dynamic process involving two conserved pectinolytic enzymes. This response integrates transcriptional changes leading to the switch from planktonic to sessile cells, a strong increase in biofilm formation, and an accelerated sporulation dynamics while conserving the potential to efficiently produce specialized secondary metabolites. As a whole, we anticipate that this response of *Bacillus* to cell wall–derived host cues contributes to its establishment and persistence in the competitive rhizosphere niche and *ipso facto* to its activity as biocontrol agent.

## Highlights

• *B*. *velezensis (B*.*v)* secretes two unique enzymes degrading homogalacturonan (HG)
• These pectinases contribute to bacterial fitness and root colonization
• HG stimulates biofilm, sporulation, and secondary metabolite production in *B*.*v*
• These traits contribute to establishment and persistence of *B*.*v* in the rhizosphere

## Graphical abstract

**Figure:**
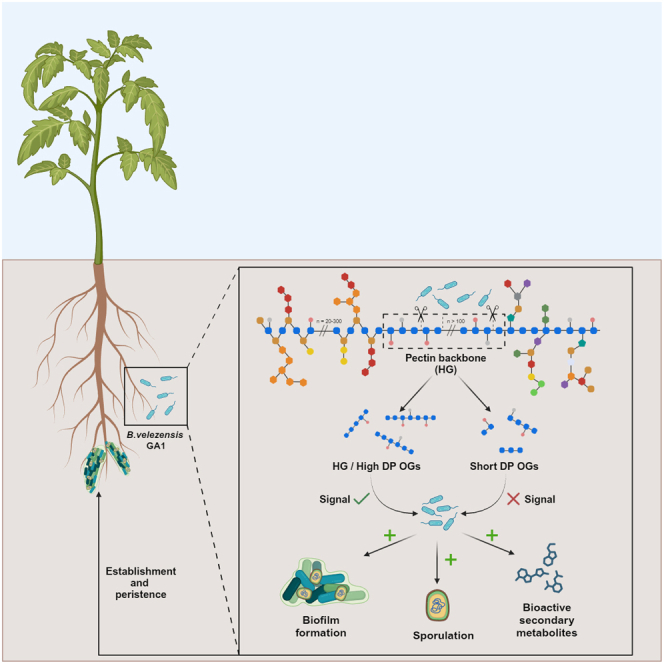

## Introduction

The rhizosphere is viewed as the narrow interface between plant roots and bulk soil directly influenced by root exudates.^1^ These root-secreted compounds represent the major nutrient source for carbon-starved soil-dwelling microbes and contribute to create a densely populated but spatially limited niche characterized by intense interspecies interactions being neutral, cooperative, or antagonistic.^2^^,^^3^^,^^4^ Root exudates thus shape the assembly of microbial communities associated with roots according to the potential of individuals to adapt to the host and to face such highly competitive context. This results in dynamic and complex rhizosphere microbiomes that comprise commensal and pathogenic but also beneficial microorganisms including bacterial species that have evolved as true mutualists establishing intricate interactions with the plant. These bacteria use root-exuded chemicals to fuel their catabolism and sustain growth while, in turn, they provide beneficial services to their host by promoting growth and/or by augmenting resistance to biotic and abiotic stresses.^5^^,^^6^^,^^7^ These beneficial rhizobacteria are usually characterized by efficient root colonization ability and stress tolerance.^8^^,^^9^ Moreover, they possess a higher number of genes involved in chemotaxis, motility, and biofilm formation compared with bacteria found in the bulk soil.^10^^,^^11^

Species of the Bacillaceae belonging to the *Bacillus subtilis* complex are common inhabitants of the rhizosphere and are among the most studied plant-beneficial bacteria (PBB), especially for their positive effects on plant health and growth.^12^^,^^13^ It includes *Bacillus velezensis*, one of the most promising rhizobacterial species to be used as biological control agent.^14^^,^^15^^,^^16^^,^^17^ This biocontrol activity mainly relies on (1) the ability to compete for space and nutrients with the other inhabitants of the rhizosphere, (2) to develop strong direct antagonism toward pathogenic (micro)organisms, and (3) to stimulate host immunity leading to induced systemic resistance (ISR).^12^^,^^18^ The expression of these traits correlates with the potential of *B*. *velezensis* to produce a wide range of chemically diverse and functionally specialized secondary metabolites (SMs) acting as signals, siderophores, antimicrobials, or immunity elicitors.^19^^,^^20^ From an ecological perspective, this arsenal is also considered as an adaptative trait to improve establishment and persistence in the competitive rhizosphere niche.

A few recent reports have described how soil bacilli and other plant beneficial species may modulate the expression of developmental traits and adapt the production of SMs upon facing microbial competitors in interspecies interactions.^21^^,^^22^^,^^23^^,^^24^^,^^25^ However, the molecular dialogue occurring with the host plant at the root level also represents a key component of the multitrophic rhizospheric interactions that may influence the behavior of these beneficials. Certain root-exuded compounds, essentially amino acids, organic acids and to a lesser extent sugars are perceived as host-derived signals by rhizobacteria which in response, move toward the host roots via chemotaxis.^26^^,^^27^^,^^28^^,^^29^ This promotes bacterial cell attachment to the root surface to initiate root colonization by forming biofilms, which are highly organized bacterial community assembly comprising vegetative cells and spores enclosed in a self-produced matrix.^25^^,^^30^ Root exudates have also been shown to modulate SMs production, among which surfactin,^31^^,^^32^ and the expression of genes involved in bacillibactin, bacilysin, bacillaene, difficidin, macrolactin, surfactin, and fengycin synthesis^33^^,^^34^ in diverse *B*. *velezensis* strains.

Another facet of the plant-rhizobacteria interaction, occurring upon direct contact with the host plant, involves the perception of plant cell wall polymers (CWPs). We and others previously showed that the production of the multifunctional lipopeptide surfactin is specifically stimulated upon sensing pectin,^35^^,^^36^ and more precisely pectin backbone,^32^ playing a key role in the first steps of root colonization. Moreover, pectin but also arabinogalactan and xylan were shown to promote biofilm formation in several bacilli species^36^^,^^37^^,^^38^ through the induction of the expression of biofilm matrix-related genes.^36^^,^^37^ In *B*. *subtilis*, the galactose contained in these polymers is used as a substrate to synthesize matrix exopolysaccharides.^37^^,^^39^ However, the phenotypic and metabolite responses of beneficial bacilli to CWPs perception still remain largely uncharacterized.

In this study, we investigated the mechanistic underlying the impact of pectin, as first CWP potentially sensed by *B*. *velezensis* upon contact with root tissues,^40^ on the expression of key developmental traits sustaining efficient colonization and on SMs production. We showed that the pectin backbone homogalacturonan (HG) acts as host-associated cue that triggers early biofilm formation, fast sporulation and enhanced production of some specialized SMs. Our data indicated that such HG sensing contributes to the establishment and persistence of *B*. *velezensis* cell populations in the rhizosphere via a dynamic process involving bacterial pectinolytic enzymes readily active *in planta*.

## Results

### Conserved polygalacturonate lyases contribute to root colonization by *B*. *velezensis*

**Figure 1:**
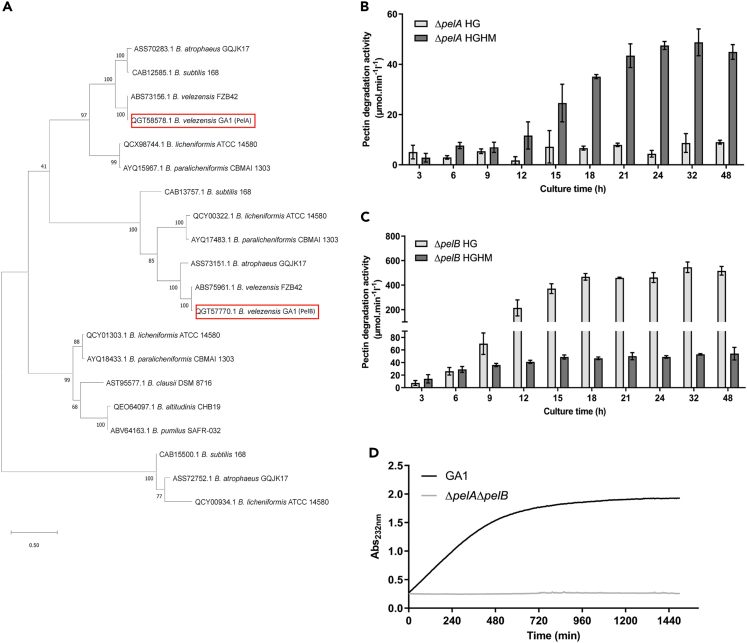
General characterization of *B*. *velezensis* pectin degrading enzymes PelA and PelB (A) Phylogenetic relationship of HG degrading enzymes between PelA and PelB of *B. velezensis* GA1 and archetype strains of other soil-dwelling bacilli. (B and C) Time-course measurement of pectin degradation activity of cell-free culture supernatants of (B) *B. velezensis* GA1 Δ*pelA* and (C) Δ*pelB* mutants on HG (light gray bars) and HGHM (dark gray bars) (Mean ± SD, n = 3). (D) Kinetics of oligogalacturonides released over time from HG by 24h-cell-free culture supernatants of GA1 (black line) and the mutant Δ*pelA*Δ*pelB* (light gray line). See also Tables S2 and S3.

In this work, the genetically amenable plant-associated strain GA1 (GenBank: CP046386.1) was used as representative of the *B*. *velezensis* species.^41^ We previously showed that GA1 possesses two genes encoding polygalacturonate lyases (*pelA*, GL331_08735; *pelB*, GL331_04125) that are conserved with a high nucleotide identity in most strains of the *B*. *velezensis* species which belong to the functional group *amyloliquefaciens* within the *B*. *subtilis* complex.^32^ These lyases act as pectin degrading enzymes by generating oligogalacturonides from pectin backbone (homogalacturonan), constituting up to 65% of total pectin encountered in the plant primary cell wall.^40^^,^^42^ Comparative genomic analysis of the enzymatic content of pectin remodeling and degrading enzymes in other soil-dwelling bacilli reveals that PelA and PelB are the only enzymes potentially involved in pectin degradation/remodeling that are produced by *B*. *velezensis* (Tables 1 and S1). On the one hand, it highlights a highly reduced pectinase arsenal in GA1 compared to other saprophytic soil species such as *B*. *subtilis*, *B*. *paralicheniformis* or *B*. *licheniformis* (Tables 1 and S1). On the other hand, none of these enzymes are synthesized by ubiquitous species such as *B*. *mycoides*, *B*. *simplex*, *B*. *thuringiensis* or *B*. *weihenstephanensis* (Table 1). We assume that such a limited potential to synthesize pectin-degrading enzymes in *B*. *velezensis* might be linked to its typical plant-associated lifestyle.^14^ In terms of phylogenetic relationship between pectinases of the PL1 and PL3 families found in these soil-dwelling bacilli, the maximum likelihood tree shows low similarity between PelA and PelB of GA1 with pectinases of the other *Bacillus* species (Figure 1A and Table S2). Moreover, PelA and PelB of *B*. *velezensis* are separated in two different clusters, suggesting notable differences in their structure and function.

**Table 1:** Content of enzymes involved in pectin degradation (polygalacturonases (PG); rhamnogalacturonan hydrolases, (RGH); polygalacturonate lyases, (PL)) and pectin remodeling (pectin methylesterases (PME) and pectin acetylesterases, (PAE)) in archetype strains of soil-dwelling bacilli

	Pectin degradation			Pectin remodeling		
	PG	RGH	PL	PME	PAE	TOTAL
*B*. *altitudinis* CHB19	1	1	2^a^	1	1	**6**
*B*. *atrophaeus* GQJK17	0	2	6^a^	0	3	**11**
*B*. *clausii* DSM8716	0	2	4^a^	0	0	**6**
*B*. *licheniformis* ATCC-14180	1	2	8^a^	1	4	**16**
*B*. *mycoides* KBAB4	0	0	0	0	0	**0**
*B*. *paralicheniformis* CBMAI1303	1	2	8^a^	1	4	**16**
*B*. *pumilus* SAFR032	1	1	2^a^	1	1	**6**
*B*. *simplex* SH-B26	0	0	0	0	0	**0**
*B*. *subtilis* 168	0	2	7^a^	0	3	**12**
*B*. *thuringiensis* CT-43	0	0	0	0	0	**0**
*B*. *velezensis* GA1/FZB42	0	0	3^a^	0	0	**3**
*B*. *weihenstephanensis* WSBC 10204	0	0	0	0	0	**0**

Enzymes are grouped by family following the classification established in the CAZy database.^104^

a means that one or several protein sequences are referred as domain of unknown function (DUF), unknown protein, hypothetical protein or putative protein. See also Table S1.

In the plant cell wall, pectin backbone is mainly low methylesterified, but it can also be found in its highly methylesterified form.^40^ Therefore, we wanted to assess whether PelA and PelB may display some substrate specificity regarding the level of methylation of the HG polymer, allowing to distinguish between pectin and pectate lyase activities.^42^ To that end, we generated and cultivated the two GA1 mutants Δ*pelA* and Δ*pelB* in a medium containing the specific sugars, organic acids and amino acids found in root exudates of Solanaceae^31^ (root exudates mimicking medium). Pel enzymes are readily produced in this medium and we measured the activity of the corresponding cell-free supernatants on commercially available HG polymer of low (≤5%, HG) and high (≥85%, HGHM) methylesterification degree. Our data reveal that PelB is moderately but exclusively active on HGHM (Figure 1B) while PelA is highly and preferentially active on HG (Figure 1C), suggesting that PelA is a pectate lyase and PelB is a pectin lyase. *In silico* analysis of structural features in the protein sequences of PelA and PelB strongly suggests that the low activity of PelB compared to PelA might be due to the absence of disordered domain(s) (Table S3), responsible of structural plasticity and proper exposition of the catalytic site for optimal functioning of the enzyme.^43^^,^^44^ Therefore, PelA and PelB thus display complementary lyase activities on HG. In addition, the double mutant *ΔpelAΔpelB* is fully impaired in its potential to breakdown HG, providing functional evidence in support to genomic data that these two Pels represent the only enzymes involved in HG degradation in GA1 (Figure 1D).

**Figure 2:**
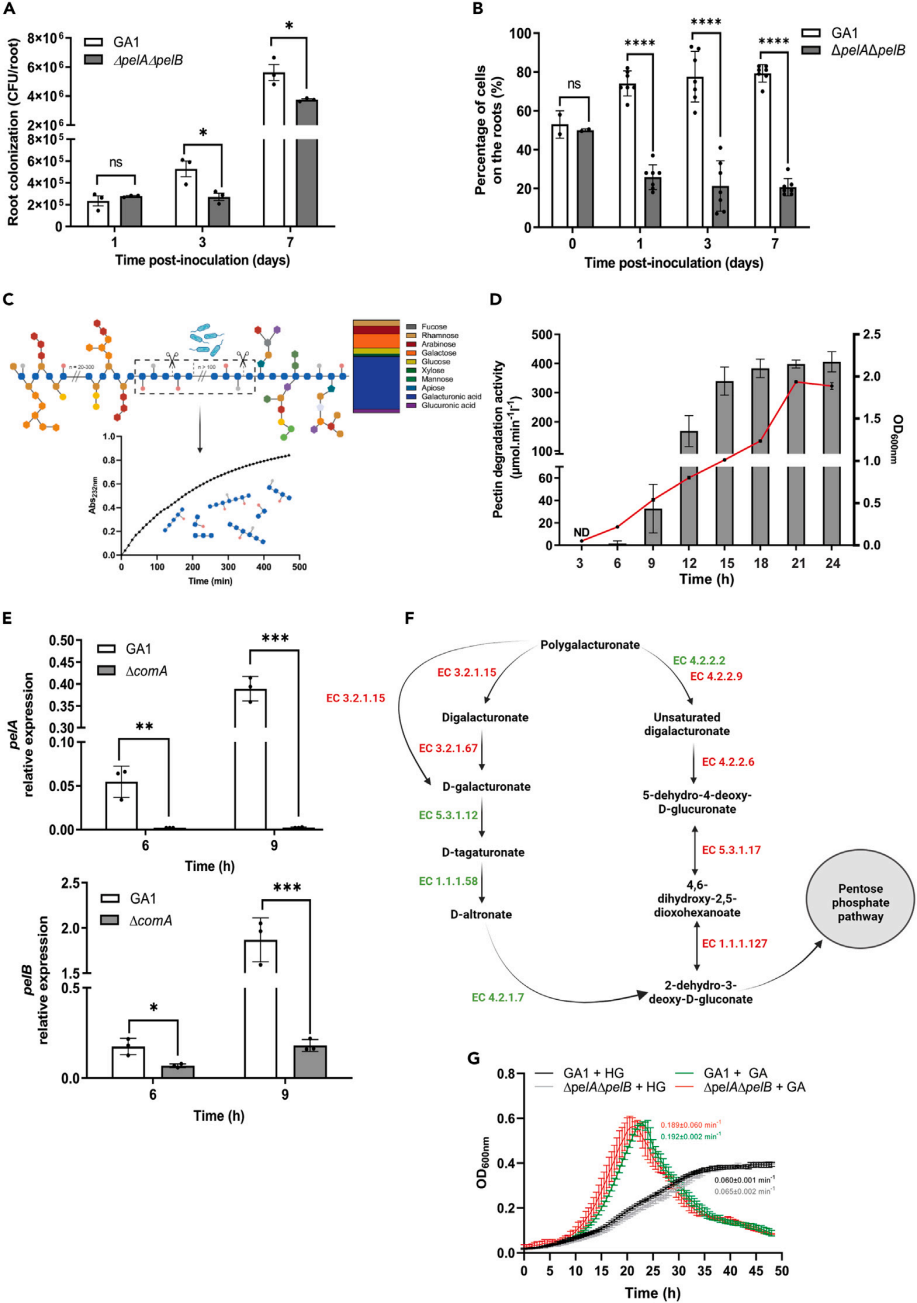
Conserved polygalacturonate lyases contribute to root colonization by *B*. *velezensis* (A and B) Time-course measurement of (A) GA1 (white bars) and Δ*pelA*Δ*pelB* (gray bars) populations on tomato plantlets roots and (B) upon co-inoculation (50:50) on tomato plantlets roots (Mean ± SD, n = 3 for (A) and n = 7 for (B), t-test; ns, non-significative; ∗, p < 0.05; ∗∗∗∗, p < 0.0001). (C) Schematic representation of pectin degradation products generated by Pels enzymes from pectin extracted from tomato plant roots (PEC, Created with biorender.com). Column chart represents simple sugar composition (expressed as molar ratio percentages) of PEC fraction. Line graph represents the kinetics of oligogalacturonides released over time from PEC fraction by cell-free culture supernatants of GA1. (D) Time-course measurement of pectin degrading activity of cell-free culture supernatants of GA1 (bars, left axis) regarding bacterial growth (line, right axis) (Mean ± SD, n = 3). (E) Expression pattern of *pelA* (up) and *pelB* (down) in GA1 (white bars) and Δ*comA* mutant (gray bars) at early growth stages (Mean ± SD, n = 3, t-test; ∗, p < 0.05; ∗∗, p < 0.01; ∗∗∗, p < 0.001; ∗∗∗∗, p < 0.0001). (F) Simplified pathway of HG degradation and assimilation (pentose and glucuronate interconversion, adapted from KEGG pathway database^47^). Enzymes encoded and not encoded in GA1 genome are marked in green and red respectively. (G) Growth profile of GA1 and Δ*pelA*Δ*pelB* mutant on minimal medium supplemented with 0.2% (w/v) HG (black and gray line respectively) or GA (green and red lines respectively) (mean ± SD, n = 3). Growth rate in each condition is indicated next to the corresponding curve and was determined by calculating the slope of the linear part of ln(OD_600_).

As mutualistic rhizobacteria, one of the key traits of *B*. *velezensis* isolates relies on their potential to efficiently colonize host roots. We previously showed that GA1 tends to form biofilm structures when colonizing tomato plantlets roots.^41^ As they represent unique functional pectin-degrading enzymes that have been conserved in the species, we postulated that PelA/B lyases may play some role in the rhizosphere fitness of the bacterium. Monitoring of bacterial population dynamics on tomato roots on solidified Hoagland medium reveals that the mutant *ΔpelAΔpelB* impaired in lyases production is also significantly affected in its colonization potential compared to the wild type strain (Figure 2A). In addition, Δ*pelA*Δ*pelB* is strongly outcompeted by GA1 upon co-inoculation at the same initial cell concentration, further indicating some reduced root fitness related to the loss of PelA/B synthesis (Figure 2B). In support to the role of these enzymes in the *Bacillus*-plant interaction *in vivo*, we detected substantial amounts of oligogalacturonides released from native pectin extracted from tomato roots (PEC) after treatment with a PelA/B-enriched extract (Figure 2C). This indicates that these enzymes may effectively alter the pectin polymer naturally found in the plant cell wall.

Based on all these data, we assumed that PelA/B are readily produced by the bacterium quite early upon root colonization. In liquid cultures, pectin degrading activity is detected as soon as GA1 cells enter into the exponential growth phase (Figure 2D), suggesting that quorum sensing might regulate their expression. This population-dependent process plays a key role in various developmental traits driving the fate of cell communities including biofilm formation and *in extenso* root colonization.^45^^,^^46^ RT-qPCR measurements of the expression levels of *pelA* and *pelB* in a *ΔcomA* mutant unable to synthesize the major regulator of quorum sensing in bacilli show that *pelA* is not expressed anymore while the expression of *pelB* is markedly repressed compared to GA1 (Figure 2E). This indicates that these enzymes also differ in their regulation in addition to differences in their activities.

Therefore, GA1 may somehow degrade to a limited extent pectin backbone during root colonization via its Pel enzymes, without impairing cell wall integrity since we never observed any adverse effect on plant health and growth caused by this beneficial bacterium. We thus hypothesized that besides root exudates, the bacterium may use oligomers of galacturonic acid (GA) generated from HG degradation as an additional carbon source to sustain growth and thereby, favor root colonization. However, according to KEGG database,^47^ the pentose and glucuronate interconversion pathway necessary for the assimilation of oligomers of GA is incomplete in *B*. *velezensis* (Figure 2F). Intriguingly, GA1 possesses the complete pathway to metabolize and use GA monomers as carbon source but is unable to generate GA monomers from HG since it does not synthesize polygalacturonases (EC.3.2.1.15) and the other required isomerases. We confirmed this experimentally since GA1 and the mutant *ΔpelAΔpelB* display a reduced but similar growth rate in minimal medium supplemented with HG while it is significantly higher in presence of GA (Figure 2G). Taking all these results together, the ability to degrade HG confers to GA1 an advantage for its establishment on roots at the early stages of colonization but without using HG degradation products as an additional carbon source.

### Homogalacturonan sensing stimulates early biofilm formation in *B*. *velezensis*

**Figure 3:**
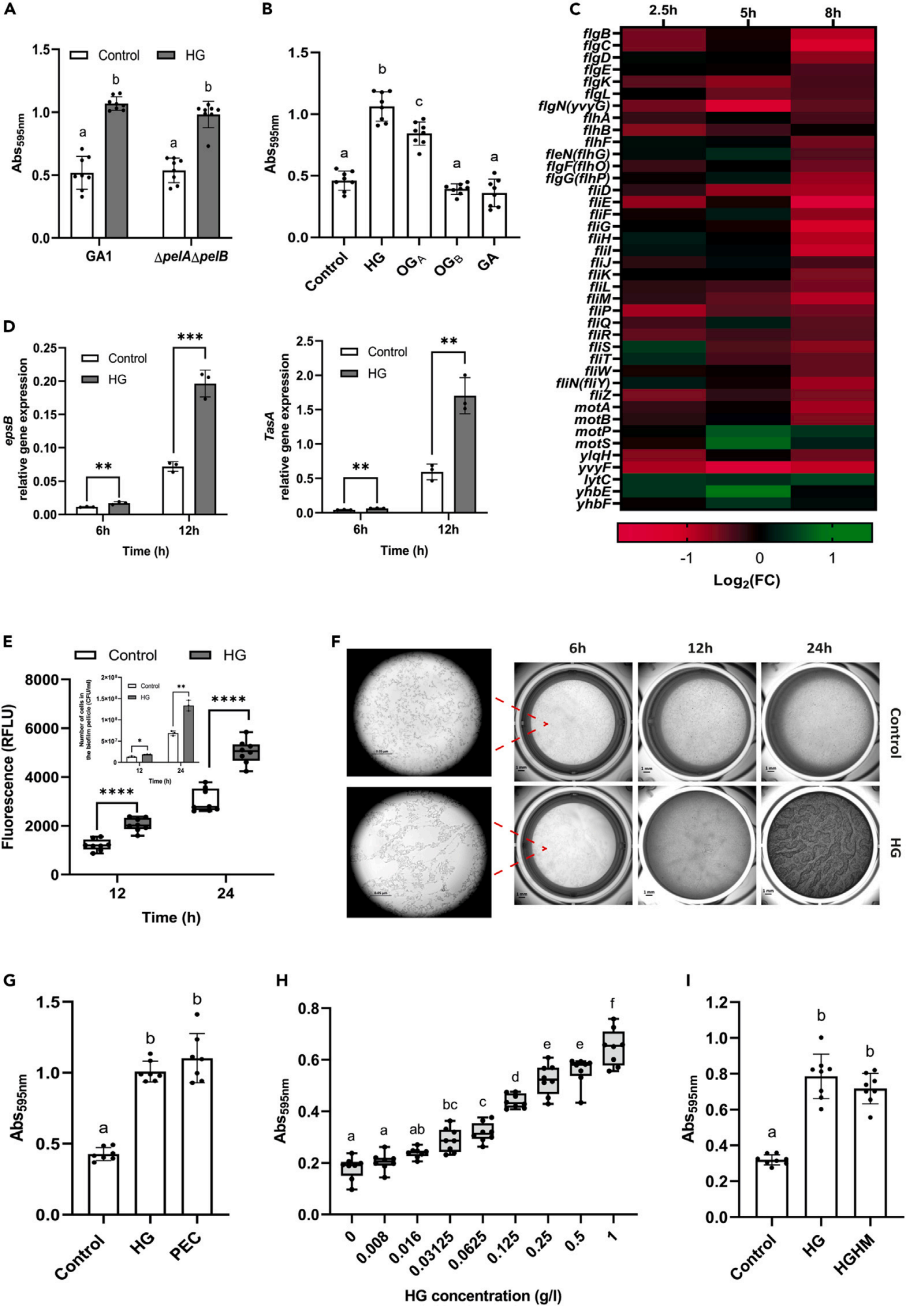
Homogalacturonan sensing stimulates early biofilm formation in *B*. *velezensis* (A and B) Assessment of biofilm formation at the air-liquid interface by (A) GA1 and Δ*pelA*Δ*pelB* in the control medium (white bars) and upon supplementation with 0.1% (m/v) HG (gray bars) in a 96-well microplate by crystal violet staining and (B) by GA1 upon supplementation with 0.1% (m/v) HG of different DP (HG, mean DP ≈ 170^32^; OG_A_, mean DP = 13; OG_B_, mean DP = 4; GA, DP = 1) (Mean ± SD, n = 8, Tukey’s multiple comparisons, α = 0.05). (C) Heatmap representing differential expression of genes involved in motility in GA1 upon supplementation with 0.1% (m/v) HG normalized to the control medium after 2.5, 5 and 8h of culture. Green and red shadings represent respectively higher and lower relative expression level compared with the control medium. (D) Expression pattern of *epsB* (left) and *tasA* (right) genes in GA1 in the control medium (white bars) and upon supplementation with 0.1% (m/v) HG (gray bars) at early biofilm formation stages (Mean ± SD, n = 3, t-test; ∗∗, p < 0.01; ∗∗∗, p < 0.001). (E) Time-course measurement of eDNA content in biofilm pellicles of GA1 formed at the air-liquid interface in the control medium (black line) and upon supplementation with 0.1% (m/v) HG (gray line) (Mean ± SD, n = 8, t-test; ∗∗∗∗, p < 0.0001). Inset: Time-course measurement of the number of cells embedded into the biofilm pellicle in the control medium (white bars) and upon supplementation with 0.1% (m/v) HG (Mean ± SD, n = 3, t-test; ∗, p < 0.05; ∗∗, p < 0.01). (F) Macroscopic observation of the kinetic of biofilm formation at the air-liquid interface by GA1 in the control medium and upon supplementation with 0.1% (m/v) HG (scale bar-1 mm). A magnification of the biofilm pellicle at 6h is shown (scale bar-0.05 µm). (G) Assessment of biofilm formation (as described in panel A and B) by GA1 in the control medium and upon supplementation with 0.1% (m/v) pectin fraction extracted from tomato plant roots (PEC) and HG (Mean ± SD, n = 8, Tukey’s multiple comparisons, α = 0.05). (H) Assessment of biofilm formation (as described in panel A and B) by GA1 upon supplementation of HG at different concentrations (Mean ± SD, n = 8, Tukey’s multiple comparisons, α = 0.05). (I) Assessment of biofilm formation (as described in panel A and B) by GA1 in the control medium and upon supplementation with 0.1% (m/v) HG and HGHM (Mean ± SD, n = 8, Tukey’s multiple comparisons, α = 0.05). See also Figures S1 and S2.

The root colonization potential of *B*. *velezensis* and related species such as *B*. *subtilis* correlates positively with their ability to form robust biofilm *in vitro* and *in vivo.*^48^^,^^49^^,^^50^^,^^51^ Therefore, we next hypothesized that enhanced fitness of GA1 during root colonization may be due to an improved potential to form biofilm in response to HG or HG degradation products. Based on measurements of biofilm pellicles at the air-liquid interface, we observed an enhanced biofilm formation in presence of HG for both GA1 and the mutant *ΔpelAΔpelB* compared to un-supplemented control conditions (Figure 3A). Furthermore, *ΔpelAΔpelB* responds optimally to undegraded HG by increasing biofilm formation but only moderately to oligogalacturonides with high degree of polymerization (DP, OG_A_). However, it does not respond to low-DP oligomers (OG_B_) or GA monomer (Figures 3B and S1Afor DP distributions of OG_A_ and OG_B_). This indicates that undegraded HG and HG degradation products of high DP are responsible for triggering biofilm formation in GA1. To further investigate the dynamics of this phenomenon, we observed the biofilm pellicle formation by microscopy at early stages. In presence of HG, bacterial cells start to agglomerate within a few hours and form long chains reducing their motility (Video S1A), while individual cells remain highly motile in control conditions 7h post inoculation (Video S1B). This earlier shift from motile to sessile behavior upon HG supplementation correlates with a global repression of genes related to flagellar protein synthesis as revealed by genome-wide RNA-seq analysis of the transcriptional reprogramming induced in GA1 within the first 8 h in presence of pectin backbone (Figure 3C).

Video S1A. Microscopic observation of early biofilm formation (at 7 h post-inoculation) of B. velezensis GA1 in microplate in the control medium, related to Figure 3

Video S1B. Microscopic observation of early biofilm formation (at 7 h post-inoculation) of B. velezensis GA1 in microplate upon supplementation with 0.1% (m/v) HG, related to Figure 3

In bacilli, biofilm initiation and maturation is related to the production of an extracellular matrix mainly composed of exopolysaccharides (EPS), cohesion proteins (TasA) and extracellular DNA (eDNA), which is essential to hold the bacterial community together within the biofilm structure.^25^ Measurements of the expression level of *epsB* and *tasA* by RT-qPCR on GA1 cells harvested from biofilm at the early stages of formation reveal an overexpression of both genes in the medium supplemented with HG compared to control conditions (Figure 3D). Our RNA-seq data also confirmed that all the genes constituting the operons responsible of EPS and cohesion protein synthesis are up-regulated in presence of HG (Figure S2A). Interestingly, among genes of the *epsA-O* operon, the protein encoded by *epsE*, in addition of being a key protein involved in EPS synthesis, has been shown to be involved in motility cessation by acting like a clutch on the flagellar motor switch protein FliG.^52^^,^^53^

On the other hand, it has been shown that the lipopeptide surfactin acts as signal triggering the expression of *eps* and *tasA* genes^54^^,^^55^ and in a previous study, we showed that the production of this compound by GA1 is stimulated in response to HG in the early exponential growth phase.^32^ Therefore, we wanted to evaluate whether this early surfactin production in presence of HG could have an impact on the enhanced biofilm formation observed here. Colorimetric quantification of biofilm formation by crystal violet staining shows that the mutant Δ*srfAA* impaired in surfactin synthesis is strongly affected in its potential to produce biofilm compared with GA1, but still displays a high responsiveness to HG regarding both pellicle formation (Figure S2B) and biofilm gene expression (Figure S2C). This indicates that surfactin synthesis stimulation by HG does not markedly contribute to the strongly enhanced biofilm phenotype triggered by the polymer in GA1.

Besides EPS and cohesion proteins, eDNA is also required at early stage of biofilm formation and contributes to its 3D architecture.^56^ Relative eDNA quantification assay shows a significantly higher eDNA content in pellicles formed in presence of HG compared with the control medium after 24h (Figure 3E), which is concomitant with a higher number of cells embedded in the biofilm pellicle (Inset Figure 3E). However, no significant differences were observed in terms of total number of cells in the wells (i.e., planktonic cells and cells embedded in the biofilm) in both conditions (approximately 4.5 × 10^8^ CFU/ml at 24h after inoculation), indicating that the accumulation of eDNA is not due to cell lysis but rather to an active secretion as reported for *B*. *subtilis*.^57^

Accordingly, both macroscopic observations and colorimetric quantification of biofilm show that GA1 pellicle starts to form earlier in presence of HG and gives rise to a thicker biofilm after 24h compared with control conditions (Figures 3F and 3G). A similar increase in biofilm formation was also measured upon addition of natural pectin extracted from tomato plantlets roots (PEC) at the same concentration (Figure 3G). In addition, we observed a significant stimulation of biofilm formation upon addition of HG at concentrations as low as 0.03 g/l (Figure 3H), suggesting the involvement of a quite sensitive perception system of the polymer in GA1. This would also discard the possibility that HG acts indirectly by creating some osmotic stress if added at sufficient concentration to the medium, which is supported by the fact that the chemical polymer PEG, added at the same concentration, does not trigger any response in GA1 (Figure S1B). That said, HG of high polymerization degree with low level of methylesterification can form egg-box structures stabilized by calcium bridges^58^ that might favor cell cohesion and contribute to biofilm architecture via a biologically unrelated process as it has been shown for cellulose in *E*. *coli* biofilms.^59^ Nevertheless, involvement of such a physico-chemical structuring effect can be ruled out since a similar increase in biofilm pellicle formation is observed upon addition of highly methylated HG, unable to form such supra-molecular conformation (Figure 3I). Collectively, these data strongly suggest that pectin perception underpins a signaling process through which bacterial cells actively detect HG fragments with a minimal length for triggering biofilm establishment.

### Homogalacturonan sensing enhances the sporulation dynamics in *B*. *velezensis*

**Figure 4:**
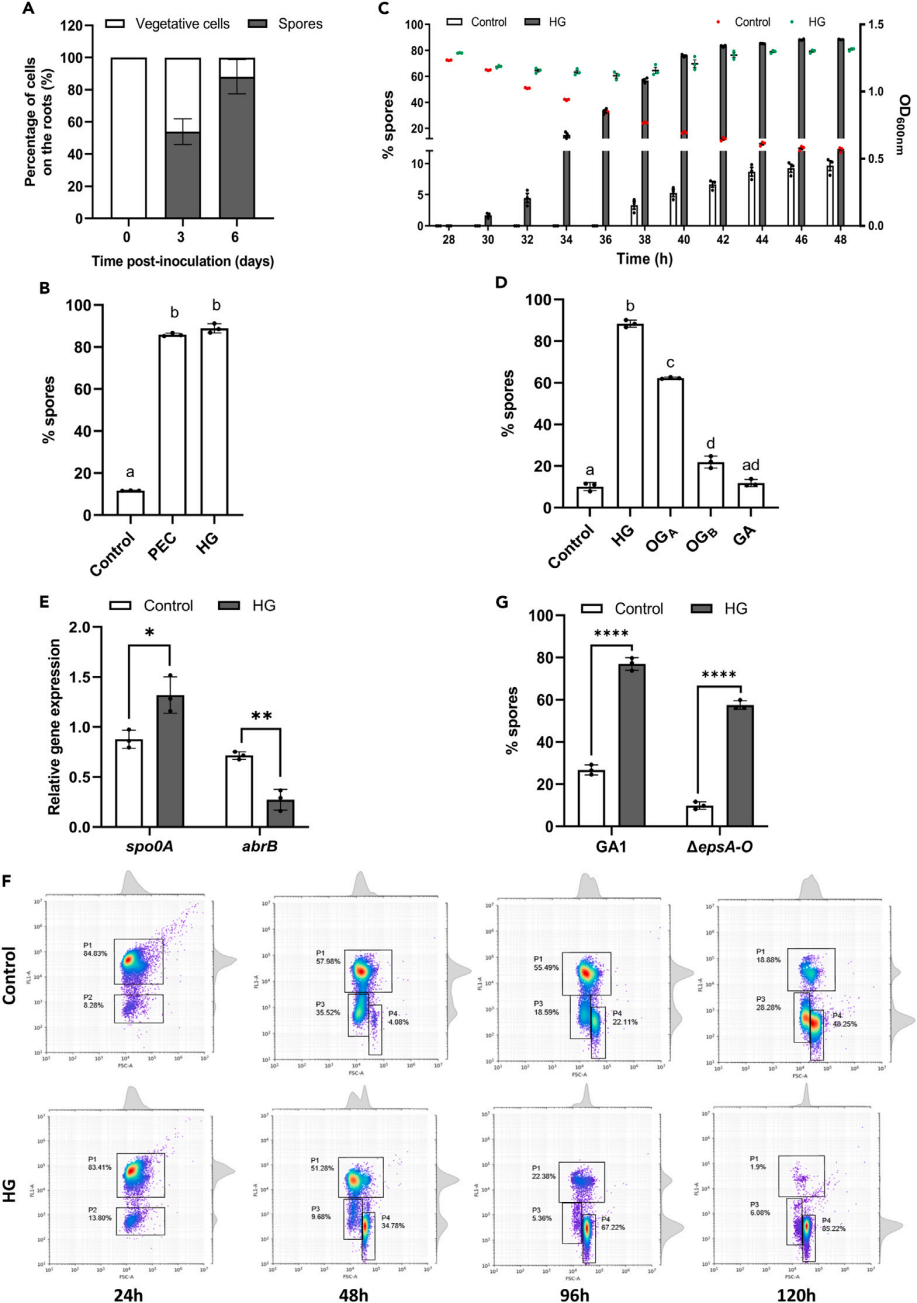
Homogalacturonan sensing enhances the sporulation dynamics in *B*. *velezensis* (A) Time-course measurement of GA1 population (vegetative cells and spores) by CFU counting upon root colonization of tomato plantlets roots (Mean ± SD, n = 6). (B) Assessment of spores percentages by flow cytometry (see STAR Methods) in planktonic cultures of GA1 after 48h upon supplementation with 0.1% (m/v) PEC or HG (Mean ± SD, n = 3, Tukey’s multiple comparisons, α = 0.05). (C) Time-course measurement of sporulation dynamics over time of GA1 (left axis) in control medium (light gray bars) and upon supplementation with 0.1% (m/v) HG (dark gray bars) regarding bacterial OD_600_ (right axis) in the control medium (red dots) and upon supplementation with 0.1% (m/v) HG (green dots) (Mean ± SD, n = 3). (D) Assessment of spores percentages (as described in Panel B) in planktonic cultures of GA1 after 48h upon supplementation with 0.1% (m/v) HG of different DP (HG, mean DP ≈ 170^32^; OG_A_, mean DP = 13; OG_B_, mean DP = 4; GA, DP = 1) (Mean ± SD, n = 3, Tukey’s multiple comparisons, α = 0.05). (E) Relative gene expression of *spo0A* and *abrB* in GA1 cells in the control medium (white bars) and upon supplementation with 0.1% (m/v) HG (gray bars) in planktonic cultures of 24h (Mean ± SD, n = 3, t-test; ∗, p < 0.05; ∗∗, p < 0.01). (F) Population diversity in biofilm pellicles formed at the air-liquid interface determined by flow cytometry after RSG staining (see STAR Methods) in the control medium and upon supplementation with 0.1% (m/v) HG. Gate P1, P2, P3, P4 stand for highly metabolically active vegetative cells, low metabolically active vegetative cells, spore-forming cells and mature spores respectively. (G) Assessment of spores percentages (as described in panel B) in planktonic cultures of GA1 and the mutant Δ*epsA-O* after 48h upon supplementation with 0.1% (m/v) HG (Mean ± SD, n = 3, t-test; ns, ∗∗∗∗, p < 0.0001). See also Figure S3.

It is commonly assumed that during biofilm formation, *Bacillus* enters into a process of cell differentiation, leading to the coexistence of vegetative cells, endospores and spores into the same multicellular structure.^60^ Within the population colonizing tomato roots, we observed that a majority of cells tends to rapidly differentiate into spores, representing more than half of the total population after 3 days and around 90% after 6 days (Figure 4A). This prompted us to also investigate the possible impact of HG recognition on sporulation as key developmental trait. We set up a method to monitor the sporulation dynamics of GA1 cells by flow cytometry using Redox Sensor Green dye (RSG) as a marker of cellular activity and cell size as proxies (See STAR Methods and Figure S3A). A strong increase in spore population is observed upon supplementation of liquid cultures of GA1 with both PEC or HG compared with the control medium after 48h (Figure 4B). We next investigated the dynamics of spore formation in GA1 population between 24h and 48h, this time frame corresponding to the end of the exponential growth phase/beginning of the stationary phase in our culture conditions. We observed that sporulation is triggered earlier and faster in GA1 cells upon HG sensing, allowing the biomass to remain constant while it sharply decreases in control conditions (Figure 4C). As observed for biofilms, the response of the bacterium regarding sporulation in presence of HG is DP-dependent. Undegraded HG and OG_A_ induce a strong increase in spore population in the *ΔpelAΔpelB* mutant while OG_B_ and GA monomer display a limited or no effect respectively compared with the control conditions (Figure 4D).

The initiation of sporulation in bacilli is regulated by the master transcription factor Spo0A, depending on its abundance and phosphorylation state.^61^ The active form Spo0A-P is indeed responsible of the direct regulation of hundreds of genes involved in the sporulation mechanism. It includes repression of the global transition state regulator AbrB, known to influence the expression of a large number of genes at late growth phase.^62^^,^^63^ We therefore anticipated that differences in expression of both *spo0A* and *abrB* genes should be induced in response to HG before the release of the first mature spores observed after 30h. Accordingly, RT-qPCR measurements show a significant increase in *spo0A* expression and a concomitant repression of *abrB* in presence of HG in cells collected after 24h (Figure 4E). The strong repression of *abrB* in presence of HG thus reflects a higher cellular concentration of Spo0A-P, necessary to initiate the sporulation mechanism.

Next, we wanted to verify if enhanced sporulation was also triggered by HG in biofilm pellicles of GA1. We analyzed by flow cytometry the population diversity of cells embedded in biofilm pellicles of GA1 and we observed that cells also sporulate faster upon HG supplementation, with a 8.5-fold increase of mature spore population in the 48h-pellicle compared with the control medium. As a consequence, the bacterial population in the mature biofilm in presence of HG is almost exclusively constituted of spores after 120h (Figure 4F). Still, such a higher sporulation dynamics could be an indirect effect of HG rather than a direct effect of its perception since it has been shown that the sporulation rate of *B*. *subtilis* NCIB 3610 increases when the bacterium produces exopolysaccharides.^64^ However, sporulation is also significantly increased in the non-EPS producing mutant *ΔepsA-O* upon HG supplementation (Figure 4G), confirming that HG perception has a direct effect on the sporulation mechanism.

### Homogalacturonan sensing impacts the production of bioactive secondary metabolites in *B*. *velezensis*

**Figure 5:**
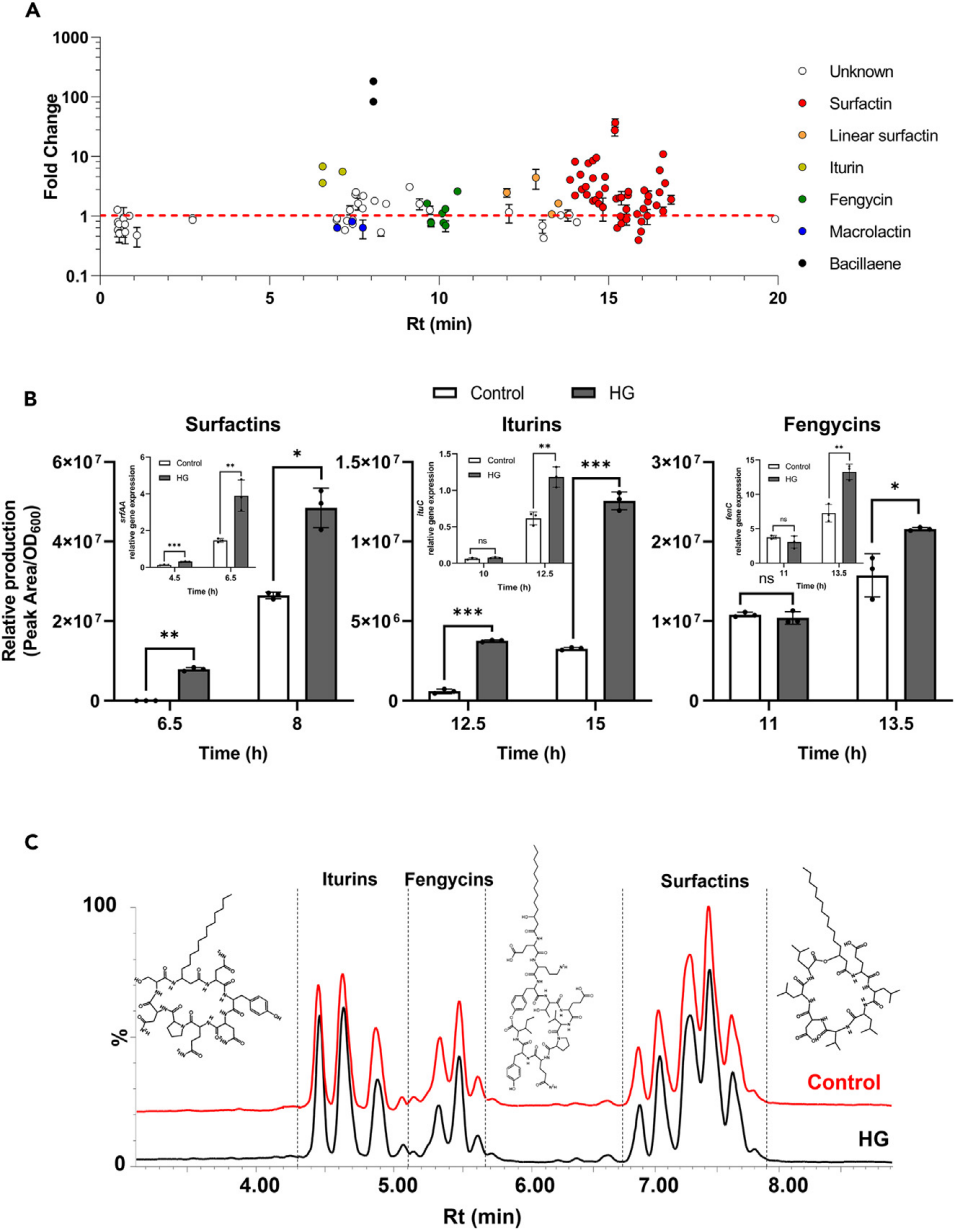
Homogalacturonan sensing impacts the production of bioactive secondary metabolites in *B*. *velezensis* (A) Impact of HG supplementation on the secondary metabolome of GA1 at late exponential/stationary phase. Each dot represents a feature detected corresponding to a specific compound (molecular ion with exact mass in UPLC-MSMS). The variety of dots in each family of metabolites reflects the natural co-production of structural variants differing in the length and isomery of the fatty acid chain and in the position of some amino acid residues in the peptide moiety. Data are expressed as peak area fold change per OD_600_, compared to control culture (Mean ± SD, n = 3). (B) Relative production of surfactins, iturins and fengycins (from left to right) in planktonic cultures of GA1 in the control medium (white bars) and upon supplementation with 0.1% (m/v) HG (gray bars) (Mean ± SD, n = 3, t-test; ns, non-significative; ∗, p < 0.05; ∗∗, p < 0.01; ∗∗∗, p < 0.001). Inset: Expression pattern of *srfAA*, *ituC* and *fenC* genes (from left to right) in planktonic cultures of GA1 in the control medium (white bars) and upon supplementation with 0.1% (m/v) HG (gray bars) (Mean ± SD, n = 3, t-test; ns, non-significative; ∗∗, p < 0.01; ∗∗∗, p < 0.001). (C) UPLC-MS total ion chromatogram (TIC) illustrating the relative abundance of lipopeptides surfactins, iturins and fengycins and their corresponding structure synthesized by GA1 cells forming biofilm at the air liquid interface in the control medium (red) and upon supplementation with 0.1% (m/v) HG (black) after 72h. The different peaks eluted for each lipopeptide family correspond to structural variants and homologs as described in panel A. See also Figure S4.

We also investigated the possible modulation of SMs production by *B*. *velezensis* in response to HG since these small-size chemicals contribute to the rhizosphere fitness of the bacterium and play key roles in its biocontrol activity.^14^^,^^15^^,^^65^ We first performed an untargeted profiling of metabolites secreted by planktonic GA1 cells in liquid cultures supplemented or not with HG. Cell-free extracts collected at stationary growth phase were analyzed by UPLC-qTOF-MS and data processing with MZmine2^66^ allowed us to detect features and the corresponding compounds differentially produced upon HG sensing. Beside unknown hits, we could identify some non-ribosomal lipopeptides (LPs) and polyketide (PKs)-type metabolites with production modulated in response to HG but in a specific way depending on the family (Figure 5A). The production of the surfactin- and iturin-type LPs and to a lower extent, the PKs bacillaene and difficidin, are significantly increased in presence of HG while fengycins and macrolactins are not or negatively impacted respectively (Figures 5A and S4A). However, due to specific transcriptional regulations, the kinetic of synthesis of these metabolites differs depending on the growth phase, which means that the amplitude and timing of the stimulating effect of HG can vary according to the molecule and sampling time. Thus, we decided to refine the time course analysis focusing on the LPs as key compounds widely produced by several plant-beneficial *Bacillus* species.^67^ We observed a pronounced early stimulation of surfactins production (Figure 5B left) as previously described,^32^ which correlates with an enhanced expression of the *srfAA* gene, one of the genes encoding for the surfactins synthetase (Inset Figure 5B left). However, our data also reveal a clear stimulation of iturins and fengycins production upon HG supplementation (Figure 5B middle and right), which is concomitant with a higher expression of the related biosynthesis genes *ituC* and *fenC* (Inset Figure 5B middle and right). This phenomenon is independent of pectin degradation products since the stimulation is also observed in cultures of Δ*pelA*Δ*pelB* cells upon HG supplementation as shown for iturins production (Figure S4B).

We also wondered whether the acceleration of sporulation dynamics in GA1 upon HG perception has an effect on the potential of the bacterium to efficiently synthesize these bioactive SMs when it forms thick spore-enriched biofilms. UPLC-qTOF-MS profiling of LPs produced by mature biofilm of GA1 with and without HG added to the medium reveal similar amounts of iturins, fengycins, and surfactins in both conditions (Figure 5C). Therefore, the faster increase in spore population in biofilm triggered upon HG perception does not impair the ability of the bacterial community to efficiently synthesize these SMs. This can be explained considering the higher production rate of iturins, surfactins and fengycins by active cells in the population even if the relative proportion of these vegetative cells is lower in presence of HG.

## Discussion

According to our current knowledge, the molecular dialogue between beneficial rhizobacteria and their host plant is mainly mediated by small-size diffusible compounds.^32^^,^^68^^,^^69^ However, signaling that may occur upon direct contact remains relatively unknown. In this work, we demonstrate that pectin backbone HG is perceived as a host cue by the beneficial species *B*. *velezensis*, which in turn, readily stimulates key developmental traits such as biofilm formation, sporulation, but also production of various bioactive SMs with specific functions. Our data come in support to the few studies mainly performed on *B*. *subtilis* reporting that some plant cell wall polysaccharides may act as trigger of biofilm formation.^32^^,^^36^^,^^37^^,^^38^^,^^39^ However, we provide a more comprehensive picture of the multifaceted response mounted by the typical plant-associated *B*. *velezensis* aiming at increasing its fitness and ensuring its persistence in its natural competitive ecological niche.

We found that the potential of *B*. *velezensis* to perceive and respond to HG is associated with its ability to secrete two pectin/pectate lyases, constituting its very limited arsenal of pectin remodeling and degrading enzymes (Tables 1 and S1). These Pels contribute to bacterial fitness since depleted mutants are significantly affected in root colonization (Figures 2A and 2B). *B*. *velezensis* can feed on galacturonic acid, the constitutive monomer of HG, only if it is already available in the rhizosphere due to its uncomplete pathway to assimilate HG degradation products (Figures 2F and 2G), in contrast with microbial plant pathogens generating and consuming monomers from HG directly encountered in the plant cell wall.^70^^,^^71^ From an ecological point of view, the presence of such enzymes in the genome of beneficials raises questions. Indeed, the ability to synthesize active plant cell wall degrading enzymes, which are very abundant in phytopathogenic microorganisms, is considered as one of the main traits responsible of their virulence.^71^^,^^72^^,^^73^ Therefore, it is tempting to speculate that such mutualistic bacteria living in intricate association with roots might have co-evolved with their host, leading to a decrease of their arsenal of pectin degrading enzymes to be tolerated by the plant. We assume that the role of Pels in colonization of plant roots by *B*. *velezensis* relies on the fact that their activity may either facilitate accessibility to the HG backbone for the bacterium without being detrimental to the host plant and release some high-DP OGs from the complex polymer, to which the bacterium is able to respond. A reduced arsenal of pectin degrading enzymes, with moderate activity, could also be used to weaken cell-to-cell adhesion by gently degrading pectin of the middle lamella to colonize intercellular spaces in root tissues as it has been shown for *Enterobacter* sp. SA187.^74^

The ability of bacteria to form robust biofilms in their natural ecological niche is considered as an essential adaptive trait. In the case of rhizobacteria, it facilitates attachment to the root and efficient colonization, which is required to exert their beneficial effects on the host plant.^25^^,^^30^^,^^50^^,^^75^^,^^76^ Here we showed that, in *B*. *velezensis*, this process is favored by the ability of the bacterium to sense HG and high-DP OGs (Figure 3B). Early biofilm pellicle formation triggered by HG (Figure 3F) correlates with transcriptional activation of genes playing key roles in biofilm initiation (i.e., EPS and cohesion proteins synthesis) and concomitant repression of genes involved in motility (Figures 3C and 3D), further illustrating the trade-off and incompatibility of both processes as reported in *B*. *subtilis*.^77^ Moreover, we detected a higher eDNA content in the biofilm pellicle upon HG sensing (Figure 3E), which can contribute to biofilm robustness since eDNA is known to interact with EPS to promote cell adhesion.^56^ Besides ensuring cell cohesion and adhesion to surfaces, which are two traits governing biofilm stability,^78^ EPS and the hydrophobin layer of the biofilm also act as a shield to protect the cell community within the biofilm from external stresses and against toxins and/or infiltration by competitors.^25^^,^^79^^,^^80^ This confers an advantage to *B*. *velezensis* in terms of ecological fitness in the highly competitive rhizosphere niche where microbial warfare dominates.

Cell differentiation is inherent to biofilm formation and guarantees the survival of the community and proliferation of the species in response to adverse abiotic conditions or microbial competitors.^81^^,^^82^^,^^83^^,^^84^ Biofilm formation and sporulation can be seen as interlinked processes since both are controlled by the same global regulators.^61^^,^^85^^,^^86^ Interestingly, our data show that the sporulation dynamics of GA1 cells is highly accelerated upon HG sensing both in biofilms and in cells in planktonic cultures (Figures 4B and 4F), independently of EPS synthesis (Figure 4G) indicating that it results directly from HG perception inducing transcriptional changes in key regulators genes of sporulation such as *spo0A* and *abrB* (Figure 4E). Via an enhanced sporulation, GA1 also avoids the strong decrease in population observed at the end of the exponential growth phase in liquid cultures (Figure 4C). Bacilli are known to exhibit a social behavior called cannibalism when they face nutrient starvation during which a portion of the population feed itself on the other to survive.^87^^,^^88^^,^^89^^,^^90^ Therefore, by enhancing sporulation dynamics upon HG perception, GA1 might highjack this phenomenon and allow a greater persistence of cells on plant roots. A prompt formation of spore-enriched biofilms should globally favor the establishment of robust populations on roots.

The secretion of SMs is also one of the factors contributing to the modulation of the fitness of the bacteria in the highly competitive rhizosphere and *B*. *velezensis* is one of the most prolific species regarding this aspect.^14^^,^^15^^,^^16^^,^^17^ In this work, we observed that HG sensing also results in modulation of the secondary metabolome of GA1 (Figures 5A, 5B, and S4A). As recently reported, it includes an increased synthesis of the cyclic LPs surfactins, favoring biofilm formation and motility and priming the plant immune system leading to systemic reinforcement of the host against phytopathogens.^32^^,^^91^ Iturins and fengycins are other lipopeptides boosted upon HG perception, mainly described for their strong antifungal activity against a wide range of phytopathogens^15^^,^^19^ but also reported as elicitors of plant systemic resistance.^92^^,^^93^^,^^94^^,^^95^ PKs synthesis of the bacillaene and difficidin families, with broad-spectrum antibiotic activities against gram-negative bacteria,^96^^,^^97^^,^^98^^,^^99^^,^^100^ is also enhanced in response to HG. Enhanced production of these SMs is thus of clear benefit for plant health and biocontrol. However, in the rhizosphere as in any other populated environment, microbes dwell in a context of competitive interactions where antibiotics are raised as weapons for chemical warfare.^12^^,^^67^ The changes occurring in the secondary metabolome described here may result in an enhanced global antimicrobial potential of *B*. *velezensis* as we reported recently upon sensing bacterial competitors in interspecies interactions.^21^ Importantly, our data also show that efficient production of these bioactive metabolites by *B*. *velezensis* is still occurring in spore-enriched biofilms (Figure 5C) and therefore in multicellular communities developing on the roots of its host on which the bacteria tend to rapidly differentiate into spores (Figure 4A).

That said, the molecular basis underpinning HG perception by bacilli cells is still unclear. Our data show that the bacterial response is not indirectly triggered by some physico-chemical cues such as structuring effect or osmotic stress that may have been caused by the addition of the polymer (Figures 3I and S1B). Moreover, *B*. *velezensis* responds to relatively low concentrations of HG (Figure 3H) but also does not react to short-DP OGs and GA monomer, suggesting some sensitive recognition system triggered selectively by high-DP HG of a minimal length. Therefore, we assume that polymer sensing may rely on a specific receptor-based recognition process as it has been reported for *Sphingomonas* spp*,*^101^ but further investigation is required to identify such putative receptor. The membrane-anchored histidine kinases (KinA-E) are good candidates since in *B*. *subtilis*, they are activated in response to a range of environmental cues including potassium leakage induced by surfactin,^22^ plant polysaccharides^37^ and potentially oxygen, light and redox potential^102^^,^^103^ which results in phosphorylation of the master regulator Spo0A acting on both biofilm formation and sporulation, according to the level of its phosphorylation state.^61^^,^^84^ However, whether such Kin system is involved or not in the perception of HG by *B*. *velezensis* remains to be established.

In conclusion, perception of HG as host-associated cue underpins a new aspect of interkingdom molecular interactions between plants and their bacterial associates such as *B*. *velezensis*. Using HG as signal allows *Bacillus* to eavesdrop its host, establish and proliferate in the rhizosphere. Improved fitness in terms of root colonization and persistence means a higher niche competition and establishment of threshold populations necessary to produce, in biologically relevant amounts, its unique arsenal of antimicrobials and plant immunity elicitors, all of them contributing to a better protection of the plant against pathogens and *in fine* to biocontrol. So, beyond the interest in microbial chemical ecology in the broad sense, this knowledge is essential for optimizing the use of *B*. *velezensis* as environmentally robust inoculant in sustainable crop production.

### Limitations of the study

In this study, we highlight the importance of HG as major constituent of pectin in the context of plant-bacteria interactions. However, the mechanism underlying the perception of this polymer as molecular pattern by *B*. *velezensis* remains to be determined, including the identification of potential specific receptor at the bacterial cell surface. Moreover, it could be relevant to study if this ability to perceive and respond to such plant CWP is also found in other bacterial species and genera commonly used as plant growth-promoting rhizobacteria (PGPR). Biocontrol assays would also be interesting to assess if the stimulation of lipopeptide production by *B*. *velezensis* upon HG supplementation may lead to higher protection of the host plant against the attack of phytopathogens. Finally, it would be relevant to extend the scope of this study and further investigate the impact of the application of exogenous HG at the plant microbiome level.

## STAR★Methods

### Key resources table

**Table:** 

REAGENT or RESOURCE	SOURCE	IDENTIFIER
**Chemicals, peptides, and recombinant proteins**		

MOPS	Sigma	Cat#M1254
Bacto™ Casamino acids	ThermoFisher Scientific	Cat#223050
Bacto™ Yeast extract	ThermoFisher Scientific	Cat#212750
HG: Galacturonan polysaccharides LM	Elicityl	Cat#GAT100
HGHM: Galacturonan polysaccharides HM	Elicityl	Cat#GAT101
GA: Galacturonic acid	Sigma	Cat#93478
OG_A_	Pr. Emmanuel Petit (UPJV, France)	N/A
OG_B_	This study	N/A
PEC	This study	N/A
Chloramphenicol	AppliChem	Cat#A1806
Phleomycin	InvivoGen	Cat#ant-ph-1
Tris-HCl	Sigma	Cat#T3253
Tween20	Sigma	Cat#P9416
TFA: Trifluoroacetic acid	Sigma	Cat#302031
Crystal Violet solution	Sigma	Cat#V5265
FloraBloom	General Hydroponics Europe (GHE)	N/A
FloraMicro	General Hydroponics Europe (GHE)	N/A
FloraGro	General Hydroponics Europe (GHE)	N/A

**Critical commercial assays**		

NucleoSpin RNA Kit	Macherey Nagel	Cat#740955.250
Luna® Universal One-Step RT-qPCR Kit	New England Biolabs	Cat#E3005L
QuantiFluor dsDNA system	Promega	Cat#E2670
*Bac*Light™ RedoxSensor™ Green vitality kit	ThermoFisher Scientific	Cat#B34954

**Deposited data**		

RNAseq data exploited in this paper	Hoff et al., 2021^32^	https://www.ebi.ac.uk/ena/ Project reference: PRJEB39762

**Experimental models: Organisms/strains**		

Bacterial strains (see Table S4)		
Tomato: *Solanum Lycopersicum* L.	Sluis Garden	Cat#SL0765

**Oligonucleotides**		

Oligonucleotides (see Table S5)	This study	N/A

**Software and algorithms**		

MEGA11	Tamura et al., 2021^107^	https://www.megasoftware.net/
MassHunter v10.0	Agilent	https://www.agilent.com/en/product/software-informatics/mass-spectrometry-software
MzMine2	Pluskal et al., 2010^66^	http://mzmine.github.io/features.html
Trimmomatic v0.39	Bolger et al., 2014^113^	http://www.usadellab.org/cms/?page=trimmomaticv
FastQC v0.11.8	Babraham Bioinformatics	https://www.bioinformatics.babraham.ac.uk/projects/fastqc/
BWA-MEM v0.7.17	Li and Durbin., (2009)^114^	https://maq.sourceforge.net/
HTSeq v0.9	Anders et al., 2015^115^	https://htseq.readthedocs.io/en/release_0.9.1/history.html#version-0-9-0
DESeq2	Love et al., 2014^117^	https://bioconductor.org/packages/release/bioc/html/DESeq2.html
NIS-Element AR software	Nikon	https://www.microscope.healthcare.nikon.com/products/software/nis-elements/nis-elements-advanced-research
GraphPad Prism 9	GraphPad Software	https://www.graphpad.com/features

**Other**		

SPARK multiplate reader	Tecan	https://lifesciences.tecan.com/multimode-plate-reader
Mixer Mill MM400	Retsch	https://www.retsch.com/products/milling/ball-mills/mixer-mill-mm-400/
Hydroponic systems	Araponics	Cat#K72L
Dionex™ ICS-3000 Ion Chromatography System	Conquer scientific	https://conquerscientific.com/product/dionex-ics-3000-ion-chromatography-system/
Dionex™CarboPac™ PA-1 column (4 mm × 250 mm)	ThermoFisher Scientific	Cat#035391
NanoDrop 2000	ThermoFisher Scientific	Cat#ND-2000
StepOne™ Real-Time PCR system	ThermoFisher Scientific	Cat#4376357
3.5kD MWCO Spectra/Por dialysis membrane	VWR	Cat#25219-085
Vivaspin20 centrifugal concentrator polyethylene sulfone 10,000Da MWCO	Sartorius	Cat#VS2001
UHPLC Agilent 1290 Infinity II	Agilent	https://www.agilent.com/en/product/liquid-chromatography/hplc-systems/analytical-hplc-systems/1290-infinity-ii-lc-system
Luna® HILIC 200 Å column (150 x 3 mm x 5 μm)	Phenomenex	Cat#00F-4450-Y0
Acquity UPLC BEH C18 column (2.1 x 50mm x 1.7μm)	Waters	Cat#186002350
6530 Q-TOF mass spectrometer	Agilent	Cat#G6530AA
Sonoplus HD 2070 ultrasonic homogenizer	Bandelin	https://profilab24.com/en/laboratory/ultrasonic/bandelin-sonopuls-hd-2070-homogeniser
BD Accuri™ C6 Plus Flow cytometer	BD Biosciences	https://www.bdbiosciences.com/en-us/products/instruments/flow-cytometers/research-cell-analyzers/bd-accuri-c6-plus
Nikon SMZ1270 stereomicroscope	Nikon	https://www.microscope.healthcare.nikon.com/products/stereomicroscopes-macroscopes/smz1270-smz1270i
Nikon Ti2-E inverted microscope	Nikon	https://www.microscope.healthcare.nikon.com/products/inverted-microscopes/eclipse-ti2-series

### Resource availability

#### Lead contact

Further information and requests for resources and reagents should be directed to and will be fulfilled by the lead contact, Marc Ongena (marc.ongena@uliege.be).

#### Materials availability

Bacterial strains and mutants generated in this study are available upon request from Marc Ongena (marc.ongena@uliege.be). This study did not generate new unique plasmids or reagents.

### Experimental model and subject details

#### Bacterial strains and construction of B. velezensis GA1 knockout mutants

All the bacterial strains used in this study are listed in Table S4. Knockout mutant strains of *B*. *velezensis* GA1 were constructed by gene replacement by homologous recombination. Briefly, a cassette containing a chloramphenicol or phleomycin resistance gene flanked by 1 kb of the upstream and downstream region of the targeted gene (see Table S5 for primers) was constructed by PCR overlap following the method developed by Bryksin and Matsumura.^105^ This recombinant cassette was introduced into *B*. *velezensis* GA1 following a slightly modified protocol developed by Jarmer et al.^106^ to induce natural competence *via* nitrogen limitation. Briefly, one colony of GA1 was inoculated in LB medium during 6h at 37°C under shaking (160rpm). Then, 1mg of the recombinant cassette was added to the GA1 washed cell suspension adjusted to an OD_600_ of 0.01 in MMG medium at pH 7 (19g/l K_2_HPO_4_, 6g/l KH_2_PO_4_, 1g/l Na_3_citrate, 0.2g/l MgSO_4_.7H_2_O, 2g/l Na_2_SO_4_, 50mM FeCl_3_ [sterilized by filtration at 0.22μm], 2mM MnSO_4_ [sterilized by filtration at 0.22 μm], 8g/l glucose, and 2g/l L-glutamic acid). Incubation was performed at 37°C under shaking (160rpm) during 24h. Cells were plated on LB medium solidified with agar (14g/l) supplemented with 5μg/ml chloramphenicol or 4μg/ml phleomycin to only select cells having integrated the recombinant cassette by double crossing over. Gene deletions were confirmed by PCR analysis with the corresponding specific upstream forward and downstream reverse primers.

#### Culture media and growth conditions

Bacterial cultures were performed at 26°C under shaking (160rpm) in root exudates mimicking medium diluted to the half (REM)^31^ at pH 7: 1g/l glucose, 1.7g/l fructose, 0.2g/l maltose, 0.3g/l ribose, 2g/l citrate, 2g/l oxalate, 1.5g/l succinate, 0.5g/l malate, 0.5g/l fumarate, 0.34g/l KH_2_PO_4_, 10.5g/l MOPS, 0.25g/l MgSO_4_ 7H_2_O, 0.25g/l KCl, 0.5g/l yeast extract, 0.5g/l Casamino acids, 1g/l (NH_4_)_2_SO_4_, 600μg/l Fe_2_(SO_4_)_3_, 200μg/l MnSO_4_, 800μg/l CuSO_4_, 2mg/l Na_2_MoO_4_. Following the experiment, PEC, HG(HM) (Elicityl), oligogalacturonides (OGs) or galacturonic acid (GA, Sigma) were normalized in terms of weight and were added at a final concentration of 0.1% (m/v) in the REM.

To assess the use of pectin backbone as a carbon source, cultures were performed in modified M9 medium (M9 stock solution adjusted to pH7: 42.5g/l Na_2_HPO_4_.2H_2_O, 15g/l KH_2_PO_4_, 5g/l NH_4_Cl, 2.5g/l NaCl; trace elements solution: 0.1g/l MnCl_2_.4H_2_O, 0.17g/l ZnCl_2_, 0.043g/l CuCl_2_.2H_2_O, 0.06g/l CoCl_2_.6H_2_O, 0.06g/l Na_2_MoO_4_.2H_2_O; Calcium chloride solution: 14.7g/l CaCl_2_.2H_2_O; Magnesium sulfate solution: 246g/l MgSO_4_.7H_2_O; Iron chloride solution: 13.5g/l FeCl_3_.6H_2_O, 21g/l citric acid.H_2_O). All the solutions were filter sterilized (0.22μm CA membrane). M9 medium was reconstituted by mixing 200ml M9 stock solution, 1ml calcium chloride solution, 10ml trace elements solution, 1ml magnesium sulfate solution, 1ml iron chloride solution, 0.1% (m/v) glycerol and the volume was adjusted to 1l with ultrapure water. The carbon sources tested were normalized in terms of weight and were added to the medium at a final concentration of 0.2% (w/v).

For planktonic and biofilm cultures, the cells of an overnight preculture of the strain of interest were washed twice with sterile phosphate-buffered saline (PBS, 8g/l NaCl, 0.2g/l KCl, 1.44g/l Na_2_HPO_4_, 0.24g/l KH_2_PO_4_; pH 7.0), before being inoculated in the liquid medium at a final OD_600_ of 0.02 (planktonic cultures) or 0.1 (biofilm cultures).

#### Plant growth conditions

For plant root colonization assays, tomato seeds were sterilized in a 70% (v/v) ethanol solution for 2min, transferred in a 20% (v/v) bleach solution under shaking during 20min, and rinsed three times with sterile water. Sterilized tomato seeds were pre-germinated during 4 days in square petri dishes containing Hoagland medium solidified with agar (14g/l) at 22°C under a 16 h/8 h day/night cycle.

To extract pectin from tomato plant roots, tomato seeds were sterilized as described above. Then, seeds were transferred into seedholders filled with Hoagland medium with 0.7% (m/v) agar and grown during 4 weeks under a 16h/8h day/night cycle in hydroponic systems (Araponics, Belgium) filled with a nutritive solution (500μl/l FloraBloom, FloraMicro and FloraGro (General Hydroponics Europe)).

### Method details

#### Phylogenetic and sequence identity analysis

All the analysis were carried out on MEGA11 software.^107^ Phylogenetic analysis was performed on a total of 20 pectin/pectate lyase sequences (classified in PL1 and PL3 family following the CAZy Database^104^) of soil-dwelling bacilli species retrieved from NCBI protein database. To construct the maximum likelihood tree, protein sequences were aligned by MUSCLE alignment and phylogenetic evolution was inferred using the “Maximum likelihood” method and “Whelan And Goldman” model (WAG) with a discrete Gamma distribution (+G). The best-fit substitution model was selected among all the substitution models implemented in MEGA11 by choosing the one yielding to the lowest BIC score for the dataset analyzed.

#### Pectin degradation activity measurement

Pectin degradation activity of cell-free culture supernatants (CFCS) of *B*. *velezensis* GA1 or GA1 knockout mutants was measured by spectrophotometry. Accumulation of unsaturated oligogalacturonates generated by the enzymes via the mechanism of β-elimination was evaluated by measuring the increase of absorbance at 232nm. Briefly, CFCS of the different strains were obtained by centrifugation (6000rpm, 10min) of the samples from the planktonic bacterial cultures in REM at different time points before being sterilized on a 0.22μm PTFE filter. Then, 25μl of the CFCS were added to 100μl of HG or HGHM (2.5g/l) dissolved in 50mM Tris-HCl buffer at pH 8. The reaction mixture was then incubated at 30°C under shaking and the increase of absorbance was measured at 232nm in a multiplate reader (Tecan SPARK, Männedorf, Switzerland). The molar extinction coefficient for unsaturated oligogalacturonates at 232nm is 4600 M^-1^ cm^-1^.^108^

#### Root colonization assay

Root colonization assays were performed on tomato plantlets under gnotobiotic conditions. 4 days-old tomato plantlets roots were inoculated with the bacterial strain of interest in the square petri dishes of solidified Hoagland medium in which the seeds were pre-germinated. To do so, cells from an overnight preculture in REM were washed twice with sterile PBS and OD_600_ was adjusted to 0.5. For the co-inoculation assay, 500μl of GA1 and Δ*pelA*Δ*pelB* cell suspensions, both at OD_600_ adjusted to 0.5, were mixed together in an Eppendorf and vortexed. Then, 5μl of the bacterial suspensions were deposited on the root top. To quantify the bacterial colonization *in-planta* overtime, the roots were separated from the aerial part of the plant after 1, 3 and 7 days of colonization. The roots were placed in tubes containing a solution of PBS supplemented with 0.1% (m/v) Tween20 and vortexed vigorously to tear off the bacterial cells from the roots. The total colony-forming units (CFUs) were determined by plating serial dilutions of the bacterial solution on solid LB medium (for GA1 cells) or solid LB medium supplemented with 5μg/ml chloramphenicol (for Δ*pelA*Δ*pelB* mutant cells) and incubated overnight at 30°C before proceeding to plate counting. For spores counting, the same solution containing the bacteria was heated at 85°C during 20min to kill vegetative cells before proceeding to the plating.

#### Extraction of pectin from tomato roots

Roots of 4 weeks-old tomato plants were harvested and lyophilized before being grinded with a Retsh MM400 mixer mill. The extraction of root cell wall was performed following an adapted protocol from Carpita^109^ and Silva et al.^110^ Briefly, 200mg of cell wall powder were resuspended into 40ml of ethanol 80% (v/v) and incubated at 90°C during 20min. After centrifugation (10,000g, 5min), supernatant was discarded and the same procedure described above was repeated 3 times on the pellet. Then, the pellet was washed with 20ml of ultrapure water and after centrifugation (10,000g, 5min), the pellet was resuspended in 10ml of acetone. The solution was centrifuged (10,000g, 5min) and the pellet was lyophilized to obtain the alcoholic residue (AR) for fractionation. The AR was resuspended in 20ml of ultrapure water and incubated at 80°C during 1h. After centrifugation (10,000g, 5min), the pellet was resuspended in 100ml of ammonium oxalate 1% (v/v) and incubated at 80°C during 2h. After centrifugation (10,000g, 5min), the supernatant was recovered, dialyzed on a 3.5kD MWCO Spectra/Por membrane (Spectrum Laboratories Inc.) and lyophilized to obtain the PEC fraction.

#### Simple sugar analysis of pectin fraction

To hydrolyse simple sugars from PEC fraction, 1ml of 2M trifluoroacetic acid (TFA) were added to 2mg of lyophilized pectin fraction and incubated at 121°C during 90min. Then, TFA was evaporated under N_2_ flow and the residue was resuspended in 1ml ultrapure water and filtered on a 0.2μm filter. Before analysis, the solution was diluted to 1mg/ml in ultrapure water. Neutral and acidic monosaccharide composition was determined by high-performance anion exchange chromatography (ICS3000 system) coupled with a pulsed amperometric detector (HPAEC-PAD) (Dionex, Thermo Scientific) equipped with a CarboPac PA-1 column (4 mm × 250 mm) and a CarboPac PA1 guard column (ID 4 mm × 50 mm). The injection volume was 25μl and elution was performed at 30°C with a constant flow rate of 1ml/min in gradient mode. For neutral sugar quantification, the mobile phases were ultrapure H_2_O (A), 160 mM NaOH (B) and 200 mM NaOH (C). The elution profiles were as follows : 0–25 min 90% A and 10% B, 25–26 min 0–100% C, 26–35 min 100% C, 25-36 min 100–0% C, 36–50 min 90% A and 10% B. For uronic acids quantification, the mobile phases were 0.16 M NaOH (A) and 0.6 M NaOAc in 0.16 M NaOH (B). The elution profiles were as follows: 0–5 min 100% A, 5–35 min 0–100% B, 35–40 min 100% B, 40–42 min 100–0% B and finally column re-equilibration by 100% A from 42 to 50 min. Monosaccharides arabinose, fucose, galactose, glucose, rhamnose, xylose, galacturonic acid and glucuronic acid (Sigma–Aldrich) were used as standards for quantification.

#### RNA extraction and RT-qPCR analysis

RNA extractions were performed with the NucleoSpin RNA Kit (Macherey Nagel, Germany), following the manufacturer’s protocol for Gram positive bacteria. The amount of RNAs extracted and their purity were assessed with the UV-vis spectrophotometer NanoDrop 2000 (Thermo scientific). RT-qPCR was performed in a ABI StepOne™ qPCR apparatus (Applied Biosystems) using the kit Luna® Universal One-Step RT-qPCR Kit (New England Biolabs, Ipswich, MA, United States). Reactions were carried out in a 20μl volume containing 10μL of Luna Universal One Step Reaction Mix, 1μL of Luna WarmStart® RT Enzyme Mix, 0.8μl of each primer (10μM) and 100ng of RNA under the following thermal cycling conditions: a 10min reverse transcription step at 55°C, a 1min initial denaturation step at 95°C and 40 cycles of 10s at 95°C and 30 s at 60°C. Melting curves were also realized from 65°C to 95°C with an increase rate of 0.5°C/5s to evaluate the specificity of the amplified products. The transcription level of genes of interest was analysed using the mathematical model proposed by Pfaffl.^111^ The *gyrA* gene encoding the DNA gyrase subunit A was used as housekeeping gene to normalise the expression level of the gene of interest.

#### Biofilm formation assay

Quantification of total biofilm was performed by crystal violet staining. Briefly, the strain of interest was inoculated in a 96-wells microplate containing 200μl of REM supplemented or not with 0.1% (m/v) HG, HGHM, OGs or GA. The plate was incubated at 30°C during 24h without shaking. Medium and planktonic cells were discarded, and wells were washed with PBS. The biofilm pellicle was stained with 0.1% (v/v) crystal violet during 10min and then wells were washed three times with PBS. The stained biofilm was dissolved with 30% (v/v) acetic acid. Absorbance was measured at 595nm.

#### Generation of short-DP OGs

OGs of short polymerization degree (OG_B_) were generated from the reaction of concentrated 24h-CFCS of *B*. *velezensis* GA1 Δ*sfp* unable to synthesize lipopeptides, on HG as substrate. Briefly, the CFCS was obtained as described in the section “pectin degradation activity measurement”. The CFCS was then concentrated 50x on a Vivaspin20 centrifugal concentrator with a polyethylene sulfone membrane of 10,000Da MWCO (Sartorius). The enzymatic reaction was performed at 30°C by mixing 25% (v/v) of concentrated supernatant with a solution of 0.5% (m/v) HG resuspended in 50mM Tris-HCl buffer at pH 8. Absorbance at 232nm was followed until the enzymatic reaction reached a plateau. Then, short-DP OGs were separated from larger molecules according to the protocol of Voxeur et al.^112^ via ethanol precipitation. The supernatant was then recovered and lyophilized.

#### Analysis of OGs profile

Relative quantification and polymerization degree of OGs were evaluated by UPLC-qTOF MS. For each sample, a volume of 10μl was injected in an Agilent 1290 Infinity II apparatus coupled with a diode array detector and a mass detector (Jet Stream ESI-Q-TOF 6530) with the parameters set up as follows: capillary voltage: 3.5kV; nebulizer pressure: 35psi; drying gas: 8l/min; drying gas temperature: 300°C; flow rate of sheath gas: 11l/min; sheath gas temperature: 350°C; fragmentor voltage: 175V; skimmer voltage: 65V; octopole RF: 750V. Accurate mass spectra were recorded in negative mode in the range of m/z = 50-1700. OGs were separated on a Luna HILIC column (3mm x 150mm x 5μm, Phenomenex, Torrance, California, USA) using a gradient of water (solvent A) and 90% acetonitrile (solvent B) that were both acidified with 0.1% (v/v) formic acid and supplemented with 15mM ammonium formate as mobile phase, with a constant flow rate of 0.5ml/min and a temperature of 40°C. For the separation of OGs, initial gradient was decreased from 100% B to 85% B in 4min before decreasing to 0% B in 16min. Solvent B was kept at 0% during 10min before going back to 100% B. Polymerization degree of the detected OGs was determined based on accurate mass measurement and relative quantification was based on peak area of the extracted ion chromatogram generated by MassHunter worksation v10.0.

#### Extracellular DNA (eDNA) detection in biofilm pellicles

Biofilms of GA1 were grown in a 96-wells black microplate containing 200μl of REM supplemented or not with 0.1%(m/v) HG and incubated at 30°C without shaking. eDNA present in the biofilm pellicles formed at the air-liquid interface was measured using the kit QuantiFluor dsDNA system (Promega, Madison, WI, USA) according to manufacturer’s protocol. Briefly, medium and planktonic cells were discarded, and wells were washed with PBS in order to only keep the biofilm pellicles. Then, 200μl of QuantiFluor® dsDNA Dye working solution (1:400 QuantiFluor® dsDNA Dye in 1X TE buffer) were dispensed into each well and homogenized with the pellicles by pipetting up and down. Microplate was incubated in the dark at room temperature during 5min and the fluorescence intensity (504nm_Ex_/531nm_Em_) was measured using a microplate reader (Tecan SPARK, Männedorf, Switzerland).

#### Cell counting in biofilm pellicles

To evaluate the bacterial population embedded in the biofilm pellicles, GA1 cells were inoculated in a 24-wells microplate containing 2ml of REM supplemented or not with 0.1% (w/v) HG and incubated at 30°C without shaking. The biofilm pellicle formed at the air-liquid interface was then collected, resuspended into 2ml PBS and mildly sonicated (Power 60%, 2 cycles, 1min) on a Sonoplus apparatus HD 2070 (Bandelin). Several dilutions were plated on solid LB medium and incubated at 30°C overnight before counting.

#### Stereomicroscopy acquisitions

Pictures of biofilm pellicles of GA1 formed at the air-liquid interface were taken with a Nikon SMZ1270 stereomicroscope (Nikon, Japan) equipped with a Nikon DS-Qi2 monochrome microscope camera and a DS-F 1× F-mount adapter. Pictures were captured in the bright field channel with an ED Plan Apo 1×/WF objective and a 0.63x magnification of with an exposure time of 40ms. Additionally, pictures of biofilm pellicles at 6h were captured with a 3x magnification and an exposure time of 10ms to get a better view of the structures in formation. Acquisitions were then processed with NIS-Element AR software (Nikon, Japan).

#### Microscopy acquisitions

Microscopy acquisitions were performed using a Nikon Ti2-E inverted microscope (Nikon, Japan) equipped with a ×20/0.45 NA S Plan Fluor objective lens (Nikon, Switzerland) and a Nikon DS-Qi2 monochrome microscope camera. Images and videos taken in the bright field channel were acquired using a Ti2 Illuminator-DIA and an exposure time of 20ms. GA1 cells strained with RedoxSenor Green (RSG) (*Bac*Light vitality kit, Thermofisher) were visualized by conventional epifluorescence microscopy with a ×100/1 oil NA S Plan Fluor objective lens (Nikon, Switzerland). A lumencor sola illuminator (Lumencor, USA) was used as the source of excitation with an exposure time of 500ms and the GFP-B HC Bright-Line Basic Filter was used. Acquisitions were then processed with NIS-Element AR software (Nikon, Japan).

#### Flow cytometry analysis

Cell activity of GA1 cells was evaluated by RSG staining. For the experiments conducted under biofilm conditions, the pellicles formed at the air-liquid interface were collected, resuspended into PBS and mildly sonicated as described in the section “cell counting in biofilm pellicles”, before staining. To stain GA1 cells, 1μl of RSG 10x diluted were added to 1ml of cell culture appropriately diluted in filtered PBS and incubated in the dark during 10min. RSG fluorescence (FL1-490nm_ex_/520nm_em_) and cell size (FSC-H) were measured by flow cytometry using a BD Accuri™ C6 appartus (BD Biosciences) under the following conditions: Medium fluidics (35μl/min); 20,000 events recorded; FSC-H threshold 20,000.

#### Analysis of BSMs

The detection of *B*. *velezensis* metabolites was performed by UPLC-qTOF MS. Before analysis, samples from planktonic cell cultures were filtered on a 0.22μm PTFE filter. For the analysis of metabolites of biofilm-forming cells, the content of each well of the microplate was mildly sonicated as described in the section “cell counting in biofilm pellicles”, before filtration. For each sample, a volume of 5μl was injected in an Agilent 1290 Infinity II appartus coupled with a diode array detector and mass detector (Jet Stream ESI-Q-TOF 6530) with the parameters set up as described in the section “analysis of OGs profile”. Accurate mass spectra were recorded in positive mode in the range of m/z =100–1700. Metabolites were separated on a C18 Acquity UPLC BEH column (2.1 × 50 mm × 1.7 μm; Waters, milford, MA, USA) using a gradient of water (solvent A) and acetonitrile (solvent B) both acidified with 0.1% formic acid as a mobile phase with a constant flow rate 0.6ml/min and a temperature of 40°C. The gradient was set up to start at 10% B and kept in this condition during 1min before raising to 100% B in 10 min. Solvent B was kept at 100% for 3.5 min before going back to the initial ratio. MassHunter v10.0 workstation and the open-source software MzMine2^66^ were used for data collection and analysis.

#### RNA-seq data analysis

The analysis of RNAseq data used in this study has been described in details previously.^32^ In the present study, we focused especially on genes involved in motility and EPS and cohesion protein synthesis. Briefly, raw RNA-seq reads were trimmed with Trimmomatic v0.39^113^ and quality control was assessed using FastQC v0.11.8 (Babraham Bioinformatics). The trimmed reads were then aligned to the genome of B. *velezensis* GA1 (GenBank: CP046386) using BWA-MEM v0.7.17.^114^ Read counts were calculated using the python-based tool HTSeq v0.9^115^ and the tables of fragments per kilobase of transcript per million mapped reads (FPKM) were generated using the Cufflinks function cuffnorm.^116^ Genes with read counts <25 were excluded. Differential expression analysis was conducted using DESeq2 pipeline,^117^ considering the following parameters: P-value<0.05 and fold change ≥1.5. Heatmaps were performed on GraphPad Prism 9.

### Quantification and statistical analyses

All statistical analyses were performed on GraphPad Prism 9. Results are expressed as mean ± SD (standard deviation). Analysis of variance (ANOVA) was performed on each data set and statistical differences between means were evaluated by two-tailed Student’s t-test or Tukey’s multiple comparisons (α=0.05). The number of biological replicates used for each experiment and P-values are indicated in the legends of the corresponding figures.

## Data Availability

• Data: The RNA-seq data sets (generated by Hoff et al.^32^) exploited for this study are deposited at https://www.ebi.ac.uk/ena/ under the project reference PRJEB39762. • *Code*: This paper does not report original code. • Any other item: Any additional information regarding the data reported in this study is available from the lead contact upon request.
