# Supplementary material for: Pectic homogalacturonan sensed by Bacillus acts as host associated cue to promote establishment and persistence in the rhizosphere
Source: iScience. 2023 Sep 15;26(10):107925. doi: 10.1016/j.isci.2023.107925 (PMC10543691; doi:10.1016/j.isci.2023.107925)
Supplement: Document S1. Figures S1–S4 and Tables S1–S5 [file mmc1.pdf]

iScience, Volume *26*

Supplemental information

Pectic homogalacturonan sensed by *Bacillus* acts as host associated cue to promote establishment and persistence in the rhizosphere

Farah Boubsi, Grégory Hoff, Anthony Arguelles Arias, Sébastien Steels, Sofija Andrić, Adrien Anckaert, Romain Roulard, Augustin Rigolet, Olivier van Wuytswinkel, and Marc Ongena

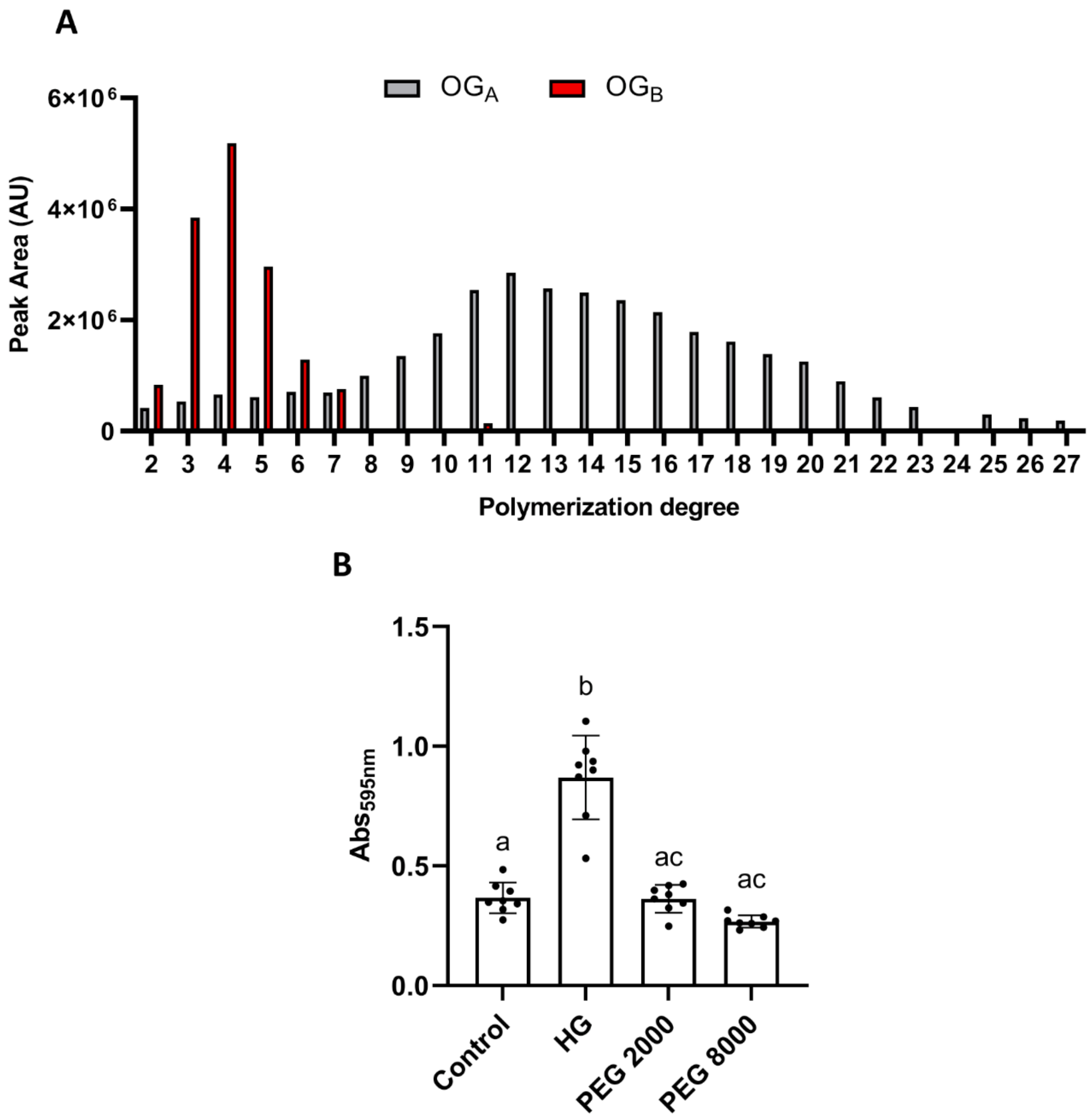

Figure S1. Stimulation of biofilm formation in *B. velezensis* GA1 upon HG sensing and impact of polymerization degree. Related to Figure 3. (A) UPLC-qTOF-MS characterization of the mean polymerization degree of OG_A (mean DP=13) and OG_B (mean DP=4). (B) Assessment of biofilm formation at the air-liquid interface by GA1 upon supplementation with 0.1% (m/v) HG and PEG of different molecular weight (PEG 2000 and 8000) in a 96-well microplate by crystal violet staining (Mean±SD, n=8, Tukey's multiple comparisons, α=0.05).

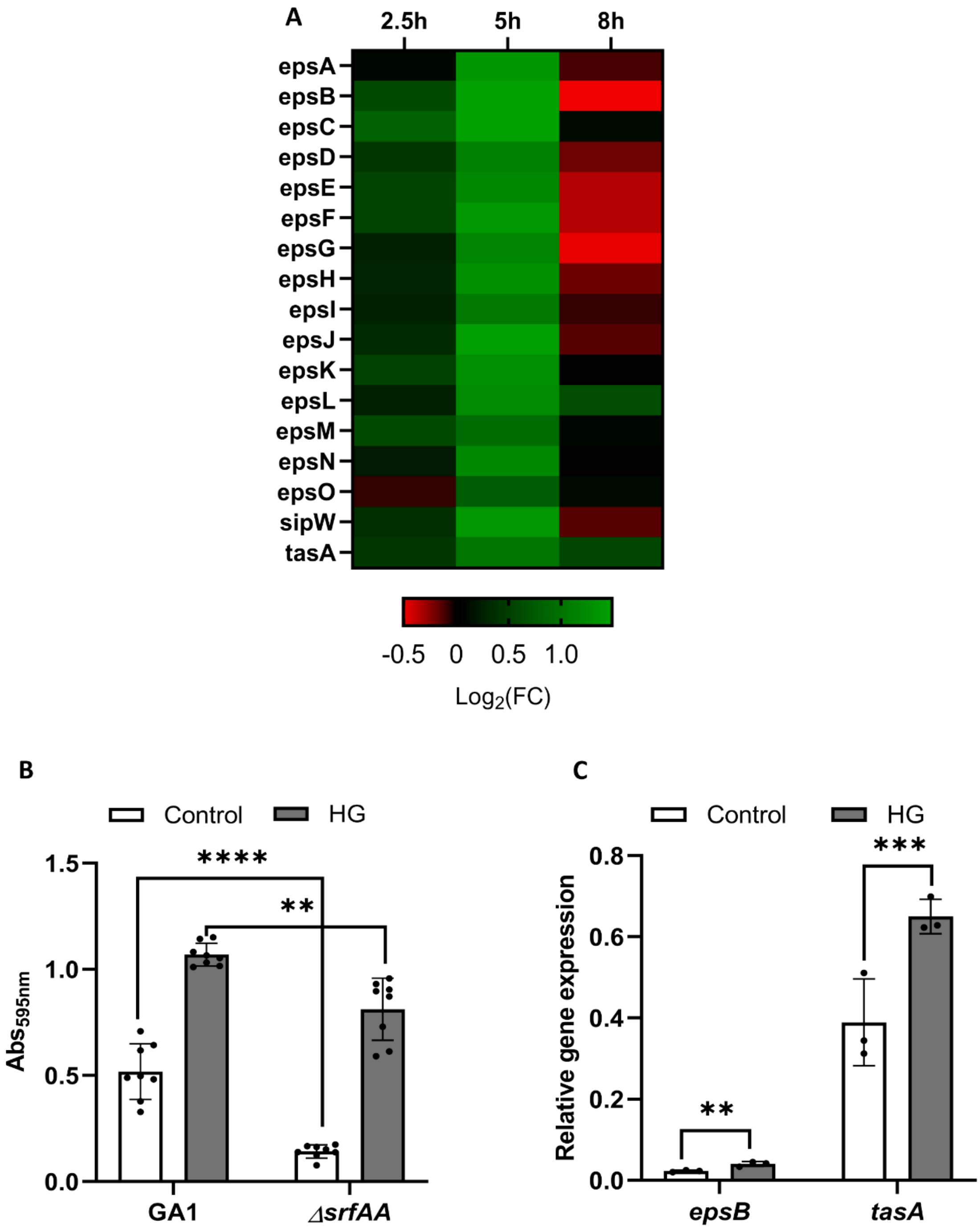

Figure S2. Implication of surfactin on EPS stimulation in *B. velezensis* GA1 upon HG sensing. Related to Figure 3. (A) Heatmap representing differential expression of genes of the epsA-O operon involved in exopolysaccharides synthesis and genes involved in cohesion protein synthesis in GA1 upon 0.1% (m/v) HG supplementation compared to the control medium after 2.5, 5 and 8h of culture. Green and red shadings represent respectively higher and lower relative expression level compared with the control medium. (B) Assessment of biofilm formation at the air-liquid interface by GA1 and the mutant Δ*srfAA* upon supplementation with 0.1% (m/v) HG in a 96-well microplate by crystal violet staining (Mean±SD, n=8, t-test; **, P<0.01; ****, P<0.0001). (C) Relative gene expression of *epsB* and *tasA* genes in Δ*srfAA* mutant cells forming biofilm in the control medium (white bars) and upon supplementation with 0.1% (m/v) HG (grey bars) after 12h (Mean±SD, n=3, t-test; **, P<0.01; ***, P<0.001).

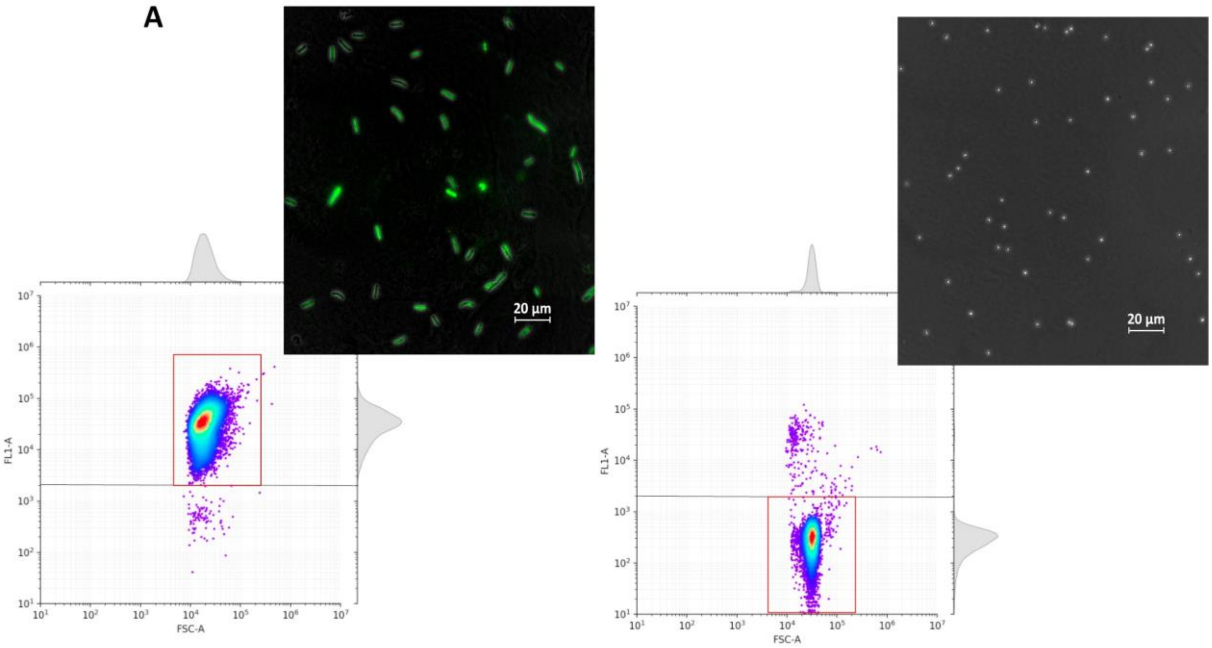

Figure S3. HG perception by *B. velezensis* GA1 stimulates sporulation. Related to Figure 4. (A) Cytograms representing fluorescence (FL1-A, Y axis) after RSG staining in function of cell height (FSC-A, X axis) of vegetative cells (left) and spores (right) of GA1. RSG being an indicator of bacterial metabolic activity, vegetative cells emit a green fluorescence contrary to spores being non-metabolically active. FL-1 threshold (horizontal line across Y axis) was set up following the autofluorescence of unstained GA1 vegetative cells. For each sample, 20,000 events were analyzed. Related microscopic observation of GA1 cells after RSG staining are shown above the cytograms.

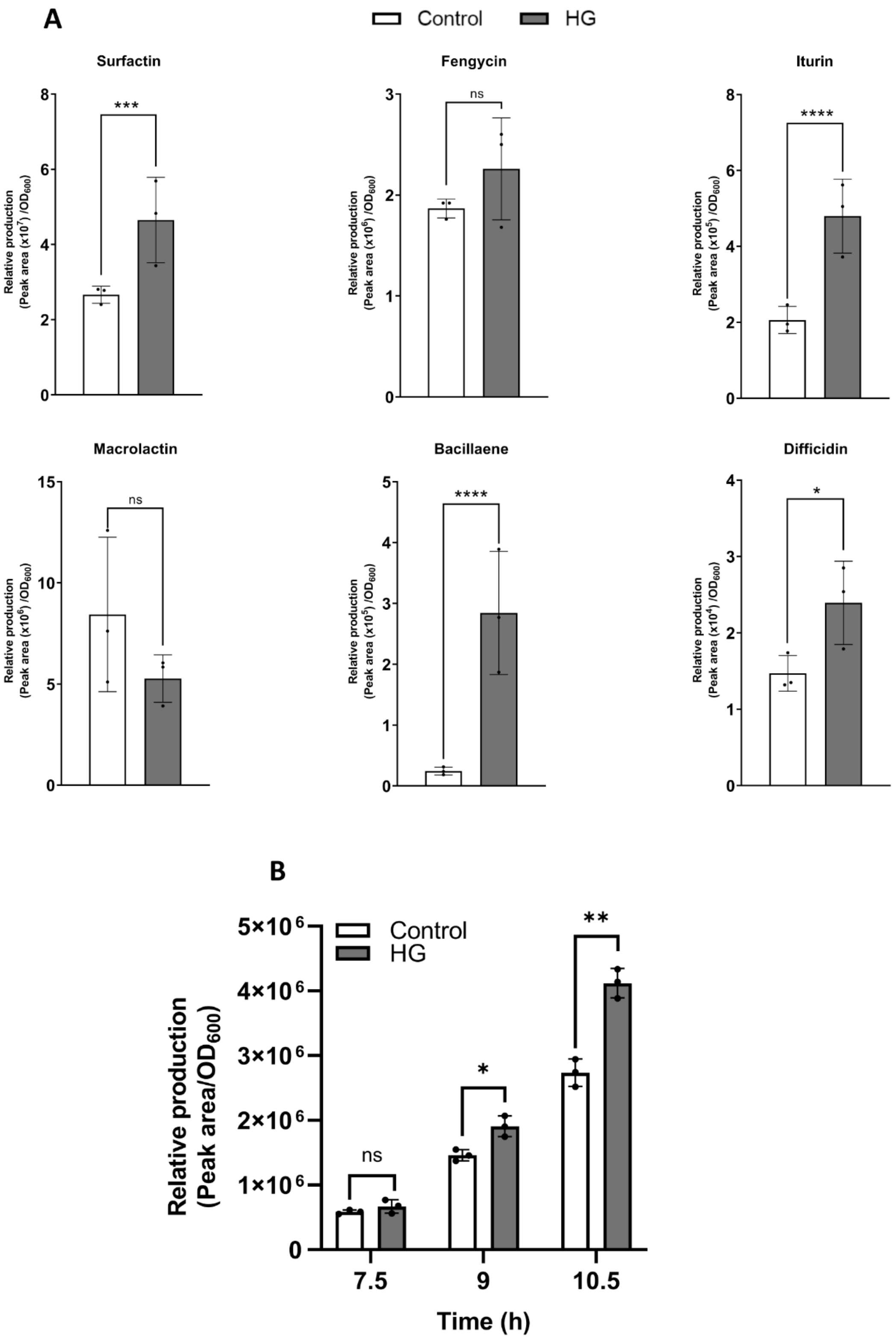

Figure S4. Production of bioactive secondary metabolites by *B. velezensis* GA1 is modulated by HG sensing. Related to Figure 5. (A) Relative production of metabolites synthesized by GA1 at late exponential/stationary phase (after 15h) in the control medium (white bars) and upon 0.1% (m/v) HG supplementation (grey bars). Peak area was normalized against the OD_{600} (Mean±SD, n=3, t-test; ns, non-significative; *, P<0.05; ***, P<0.001; ****, P<0.0001). (B) Relative production of the lipopeptide iturins by ΔpelAΔpelB in the control medium (white bars) and upon supplementation with 0.1% (m/v) HG (grey bars). Peak area was normalized against the OD_{600} (Mean±SD, n=3, t-test; *, P<0.05; **, P<0.01).

		***B. altitudinis* CHB19**	***B. atrophaeus* GQJK17**	***B. clausii DSM* 8716**	***B. licheniformis* ATCC 14580**	***B. paralicheniformis* CBMAI 1303**	***B. pumilus* SAFR 032**	***B. subtilis* 168**	***B. velezensis* GA1/FZB42**
**Polygalacturonases**	GH28	EVS87_015815	/	/	EJ992_17600	D5285_18775	BPUM_2983	/	/
	GH78	/	/	/	/	/	/	/	/
**Rhamno-galacturonan hydrolases**	GH105	EVS87_014190	BaGK_04285	BC8716_00390	EJ992_15935	D5285_07570	BPUM_2659	BSU3012	/
		/	BaGK_15610	/	EJ992_06960	D5285_16600	/	BSU07000	/
		/	/	/	/	/	/	/	/
	GH106	/	/	BC8716_21425	/	/	/	/	/
**Polygalacturonate lyases**	PL1	EVS87_018355	BaGK_04525	BC8716_06235	EJ992_07125	D5285_07740	BPUM_3515	BSU07560	RBAM_007720
		/	BaGK_20450	/	EJ992_15405	D5285_16075	/	BSU18650	RBAM_036320
		/	/	/	EJ992_20660	D5285_21390	/	/	/
	PL3	/	BaGK_18285	/	EJ992_18735	/	/	BSU34950	/
	PL4	/	/	/	/	/	/	/	/
	PL9	EVS87_018280	BaGK_12140	BC8716_11455	EJ992_10575	D5285_10745	BPUM_3499	BSU36460	RBAM_032390
	PL10	/	/	BC8716_00430	/	/	/	/	/
	PL11	/	BaGK_04310	BC8716_04435	EJ992_06985	D5285_07595	/	BSU07050	/
		/	/	/	EJ992_07000	D5285_07610	/	BSU07060	/
	PL26	/	BaGK_04335	/	EJ992_07020	D5285_07630	/	BSU07090	/
**Methylesterases**	CE8	EVS87_009500	/	/	EJ992_17535	D5285_18700	BPUM_1808	/	/
**Acetylesterases**	CE12	EVS87_003835	BaGK_04295	/	EJ992_06995	D5285_07580	BPUM_0776	BSU07020	/
		/	BaGK_04325	/	EJ992_06970	D5285_07605	/	BSU07070	/
		/	BaGK_20390	/	EJ992_07010	D5285_07620	/	BSU39120	/
		/	/	/	EJ992_20515	D5285_21470	/	/	/

Table S1. Related to Table 1. Detailed content of enzymes involved in pectin degradation and remodelling following the classification established in the CAZy database[S1] in archetype strains of other soil dwelling bacilli. Protein accession numbers in red, blue, green and yellow indicate domain of unknown function (DUF), unknown protein, hypothetical protein or putative protein respectively.

	QGT58578.1 PelA (*B.vel*)	QGT57770.1 PelB (*B.vel*)
	% Identity	% Identity
**QGT58578.1 PelA (*B.vel*)**	100%	38.95%
**QGT57770.1 PelB (*B.vel*)**	38.95%	100%
**QEO64097.1 (*B.alt*)**	44.18%	54.33%
**QCY01303.1 (*B.lich*)**	45.33%	52.53%
**QCY00934.1 (*B.lich*)**	35.84%	47.79%
**QCY00322.1 (*B.lich*)**	17.02%	50.57%
**QCX98744.1 (*B.lich*)**	64.48%	36.82%
**CAB15500.1 (*B.sub*)**	36.17%	47.62%
**CAB13757.1 (*B.sub*)**	41.57%	71.68%
**CAB12585.1 (*B.sub*)**	83.63%	38.29%
**AYQ18433.1 (*B.plich*)**	45%	52.37%
**AYQ17483.1 (*B.plich*)**	17.51%	51.22%
**AYQ15967.1 (*B.plich*)**	64.32%	36.66%
**AST95577.1 (*B.cl*)**	44.84%	54%
**ASS73151.1 (*B.at*)**	38.29%	89.68%
**ASS72752.1 (*B.at*)**	36.17%	47.95%
**ASS70283.1 (*B.at*)**	82.32%	37.64%
**ABV64163.1 (*B.pum*)**	44.18%	54.17%
**ABS75961.1 (*B.vel*)**	39.27%	98.85%
**ABS73156.1 (*B.vel*)**	99.34%	38.78%

Table S2. Related to Figure 1. Comparison of the percentage identity of pectin/pectate lyases amino acid sequences (PL1 and PL3 family) of archetype strains of other soil dwelling bacilli with PelA and PelB of *B. velezensis* GA1. *B. alt = B. altitudinis*; *B.lich = B. licheniformis*; *B.sub = B. subtilis*; *B.plich = B. paralicheniformis*; *B.cl = B. clausii*; *B.at = B. atrophaeus*; *B.pum = B. pumilus*; *B.vel = B. velezensis*.

	PelA (QGT58578.1)	PelB (QGT57770.1)
**Lenght**	421 amino acids	354 amino acids
**Superfamily**	Pectate lyase-like β-helix (CL0268)	Pectate lyase-like β-helix (CL0268)
**Domain(s)**	Pectat_lyase_4 (PF00544) in region 129-346	Pectat_lyase_4 (PF00544) in region 101-283
**Active site**	Predicted at residue 301	Predicted at residue 244
**General structure**	Right-handed β-helix	Right-handed β-helix
**Topological domain(s)**	Non-cytoplasmic in region 22-420<br>Peptide signal in region 1-21	Non-cytoplasmic in region 33-354<br>Peptide signal in region 1-32
**Disordered region(s)**	One in region 120-147<br>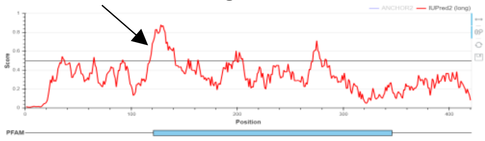	None<br>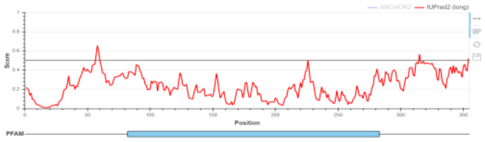

Table S3. Related to Figure 1. In silico comparison of general structure features of PelA and PelB. Protein sequences were analyzed with the progiciel HMMER[S2], the predictor IUPred2A[S3] and the web interface Phobius[S4].

**Strain name**	**Genotype**	**Reference**
GA1	Wild type (CP046386)	[S5]
Δ*comA*	GA1 Δ*comA*::cat; Chl^+	This study
Δ*srfAA*	GA1 Δ*srfAA*::cat; Chl^+	[S6]
Δ*sfp*	GA1 Δ*sfp*::cat, Chl^+	This study
Δ*pelA*	GA1 Δ*pelA*::cat, Chl^+	This study
Δ*pelB*	GA1 Δ*pelB*::phl, Phl^+	This study
Δ*pelA*Δ*pelB*	GA1 Δ*pelA*::cat Δ*pelB*::phl, Chl^+ , Phl^+	This study
Δ*epsA-O*	GA1 Δ*epsA-O*::cat, Chl^+	This study

Table S4. Related to STAR Methods. Bacterial strains used in this study.

KO mutants		
**Targeted gene**	**Primer name**	**Primer Sequence (5'→3')**
comA	comAUpFw	TCAAGTGCTTTTAAATAGTGTTACTGT
	comAUpRv	CAGGAAACAGCTATGACTTTCATGGTTTCCTCCCTTTT
	comAdwFw	GTAAAACGACGGCCAGTAACGTACTATAAATCTTGAAGGGAGAA
	comAdwRv	CTTGATGGGATCGGCATT
sfp	sfpUpFw	TCGTCACCCATGAAATCAAA
	sfpUpRv	CCAATTTTCGAATTCTTTTACCGCGCATGTCCAGATCCTCCGTCT
	sfpDwFw	CAGCTCCAGATCCTCTACGCCGGACGACGGGATTGAGATGAAAA
	sfpDwRv	CATTGAGACGTACCCGCTTT
pelA	pelAUpFw	TTCATAGGCATTCGTTTTTGAA
	pelAUpRv	CAGGAAACAGCTATGACCATGAATTGTGCTCCTCCTG
	pelADwFw	GTAAAACGACGGCCAGTTAATAAACAAGAAGCACAAAGGAA
	pelADwRv	CCTGTGACAAAAGCAAAACG
pelB	pelBUpFw	TGCAAGCTGAACAAACATCA
	pelBUpRv	TTACCGCGCAGGAAACAGCTATGACCTTTTTCATGCCCTTCACTCC
	pelBDwFw	GTAAAACGACGGCCAGTTATCCATAATACGAAAAACCG
	pelBDwRv	TCTAAACGTATCCTGTCTTGA
epsA-O	epsA-OUpFw	CGGGCAAATAGAGCCGTACG
	epsA-OUpRv	CAGGAAACAGCTATGACATTCTCATTCATGTAATTAC
	epsA-ODwFw	GTAAAACGACGGCCAGTCTTTGAATGAGCGGAAGGTT
	epsA-ODwRv	AGCTGGATGTCAGGAAACGA
**Antibiotic markers**		
Chloramphenicol	catFw	CGCGGTAAAAGAATTCGAAAA
	catRv	GTCCGGCGTAGAGGATCTG
Phlemoycin	PhleoFw	GTCATAGCTGTTTCCTGCCAAAAGGGGGTTTCATTTT
	PhleoRv	ACTGGCCGTCGTTTTACTCCAATAAATGCGACACCAA

Table S5. Related to STAR Methods. Primers used in this study.

Supplemental references

S1. Cantarel, B.L., Coutinho, P.M., Rancurel, C., Bernard, T., Lombard, V., and Henrissat, B. (2009). The Carbohydrate-Active EnZymes database (CAZy): an expert resource for Glycogenomics. Nucleic Acids Res. *37*, D233–D238. 10.1093/nar/gkn663.

S2. Finn, R.D., Clements, J., and Eddy, S.R. (2011). HMMER web server: interactive sequence similarity searching. Nucleic Acids Res. *39*, W29–W37. 10.1093/nar/gkr367.

S3. Mészáros, B., Erdős, G., and Dosztányi, Z. (2018). IUPred2A: context-dependent prediction of protein disorder as a function of redox state and protein binding. Nucleic Acids Res. *46*, W329–W337. 10.1093/nar/gky384.

S4. Käll, L., Krogh, A., and Sonnhammer, E.L.L. (2004). A Combined Transmembrane Topology and Signal Peptide Prediction Method. J. Mol. Biol. *338*, 1027–1036. 10.1016/j.jmb.2004.03.016.

S5. Toure, Y., Ongena, M., Jacques, P., Guiro, A., and Thonart, P. (2004). Role of lipopeptides produced by *Bacillus subtilis* GA1 in the reduction of grey mould disease caused by Botrytis cinerea on apple. J. Appl. Microbiol. *96*, 1151–1160. 10.1111/j.1365-2672.2004.02252.x.

S6. Hoff, G., Arguelles Arias, A., Boubsi, F., Pršić, J., Meyer, T., Ibrahim, H.M.M., Steels, S., Luzuriaga, P., Legras, A., Franzil, L., et al. (2021). Surfactin Stimulated by Pectin Molecular Patterns and Root Exudates Acts as a Key Driver of the *Bacillus* -Plant Mutualistic Interaction. MBio *12*, e0177421. 10.1128/mBio.01774-21.
